# Optimization of compact fractal monopole antenna with partial fractal ground using machine learning approach for multiband applications

**DOI:** 10.1038/s41598-025-26143-5

**Published:** 2025-11-25

**Authors:** Guntamukkala Yaminisasi, Pokkunuri Pardhasaradhi, Satti Sudha Mohan Reddy, Kokku Aruna Kumari, Om Prakash Kumar, Ishwar Bhiradi, B. T. P. Madhav

**Affiliations:** 1 Department of Electronics and Communication Engineering, Koneru Lakshmaiah Education Foundation, Vaddeswaram, Guntur, 522502 Andra Pradesh India; 2 Department of ECE, SRKR Engineering College, Bhimavaram, India; 3 CSE Department, SRKR Engineering College, Bhimavaram, AP India; 4 Department of Electronics and Communication Engineering, Manipal Institute of Technology, Manipal Academy of Higher Education, Manipal, 576104 India; 5 Department of Mechatronics, Manipal Institute of Technology, Manipal Academy of Higher Education, Manipal, 576104 India

**Keywords:** Compact microstrip antenna, Gaussian process regression, Support vector regression, Machine learning, Multiband operation, Antenna optimization, Electromagnetic simulation, SDG 9 (Industry, Innovation and infrastructure) and SDG 12 (Responsible consumption and production), Engineering, Optics and photonics, Mathematics and computing, Computer science

## Abstract

In this research, we investigate the integration of machine learning techniques, in particular Gaussian Process Regression (GPR) and Support Vector Regression (SVR), into the optimization of compact microstrip antenna design. Multiband operation with a significant miniaturization is achieved by proposing a unique circular radiating structure with decorative slots and a central star shaped patch. GPR and SVR models were used to predict and optimize critical antenna parameters such as resonant frequency, slot dimensions and patch dimensions. GPR gave better prediction accuracy with an MSE of 0.15, a score of 0.98 and takes longer wall time to converge, while compared to SVR model it converged faster with an MSE of 0.20, and a score of 0.95. The results were validated by close agreement between simulated and measured results, and the optimized design exhibited multiband performance across VHF, UHF, L, S, and C bands. These findings show that machine learning can offer a scalable and efficient alternative to the traditional methods in antenna design. With this approach, it is possible to lower the level of computational effort needed in traditional design methods.

## Introduction

The need for compact, efficient and high-performance antennas has increased rapidly with the ever-growing demand for wireless communication technologies. However, microstrip antennas have been widely studied due to their inherent advantages such as low profile, lightweight, easy fabrication and compatibility with modern electronic circuits^1^. These attributes make microstrip antennas highly desirable for applications in mobile communications, satellite systems and wearable devices However, the design of such high performance compact microstrip antennas (e.g., wide bandwidth, high gain, multiband operation) is difficult^2^ because of the complex interdependencies between geometrical and material parameters.

In the recent years, Machine Learning (ML) has been shown as a new innovative tool for antenna design, owing to the development of computational techniques^3^. In contrast, ML models and optimizes nonlinear high dimensional systems to reduce computational burden of the traditional trial and error simulation and offer predictive insights. A particularly powerful ML technique for antenna design has been GPR, a non-parametric Bayesian approach. This can handle small datasets and quantifies uncertainty in predicting critical parameters such as resonant frequency, bandwidth and radiation efficiency^4,5^.

In this research, a novel GPR based compact microstrip antenna design is proposed, optimizing its key physical and electrical parameters. A distinctive circular radiating structure with decorative slots is proposed as the antenna, including a central star shaped patch. This innovative geometry is also multiband capable, supporting VHF, ULF, L, S, and C band operation. By integrating GPR into the design process, which can predict very precisely performance metrics and reduce the need for extensive electromagnetic simulations and iterative prototyping. Frequently, multiband designs are endorsed in situations where different bands are needed for services (e.g., telemetry, communication and navigation), because having multiple small bands tends to be less power-consuming and less prone to interference than a wide single band. Slots are cut into the circular panels, so the current travels different routes and produces variety in resonance. Each hole or indent in the mask helps achieve the needed multipoint tuning. Such capacitance and inductive effects come from the etched slot dimensions and their arrangement.

The objective of this work is to design and optimize the compact fractal monopole antenna that has multi band performance. Parametric optimization is done for enhanced bandwidth and radiation efficiency. Different ML algorithms are employed to predict antenna parameters thereby reducing the number of EM simulations and demonstrating that the proposed one provides efficient alternative than the conventional antenna design and optimization techniques reducing the complexity.

This paper is structured as follows: Sect. 2 introduces the modelling approach and discuss the antenna design, and the methodological framework used. Results and a detailed discussion on simulated and measured performance metrics are presented in Sect. 3. Section 4 concludes the research with a discussion of its contributions and prospects for ML driven antenna design.

Podder et al. reviewed comprehensively the application of machine learning (ML) and deep learning (DL) in the antenna design, optimization and choice. In this work, they studied how ML models can enhance the performance of antenna systems in solving complex design challenges, like multiband and MIMO antennas. In this review, the integration of DL techniques for automatic feature extraction for improved prediction and optimization in different antenna configurations was reviewed. The results show that ML and DL can significantly decrease computational costs and increase accuracy of antenna design^6^. For multi band MIMO microstrip antennas in 5G mm Wave applications, Chbeine et al. propose an AI driven design method. The results from the study showed that ML algorithms can lead to substantial gain and efficiency improvements of the antenna geometry. The method was validated through simulations and prototypes, and it was shown that the methodology has the potential to address high frequency challenges. The designs of the next generation of antennas for the next generation communication system are shown to be possible using AI^7^.

Yusuf et al. present the design of frequency reconfigurable compact microstrip patch antennas based on a machine learning approach, where ML algorithms were used to tune bandwidth and resonant frequency, with the optimal configurations. This methodology reduced the dependence on traditional simulation driven iterations by extensive training of data to predict antenna performance metrics with high precision. Future research in adaptive and reconfigurable antenna systems^8^ relies on the work. Raveendra also optimized the microstrip antenna arrays for 5G sub 3.5 GHz networks. We applied ML techniques to optimize sequential rotation-based MIMO configurations to increase network performance and reduce interference. These results are also useful for designing robust and efficient antenna systems for next generation communication networks in the sub 6 GHz spectrum^9^.

Machine learning models were then applied to predict the dimensions of the rectangular patch microstrip antenna by Kurniawati. We demonstrated that critical design parameters (patch length and width) can be accurately determined from target performance metrics using ML based approaches^10^. Using machine learning models, Jain et al. have improved performance of circular microstrip patch antennas. The study identified and optimized the critical design parameters, slot dimensions and substrate properties that maximize gain and bandwidth. The complex design spaces were shown to be successfully explored by ML, which led to better antenna performance^11^. They also showed how ML techniques are suitable for a broad range of antenna configurations and performance requirements^12^. Deep kernel learning is used by Shudan et al. for resonant frequency modelling of micro strip antennas. The study demonstrates the potential of advanced ML models to solve complex design challenges and achieved high accuracy and computational efficiency. Their findings demonstrate the applicability of kernel-based learning techniques to antenna parameter optimization. Mohammadi et al. designed a 1 × 4 microstrip antenna array on human thigh for gain enhancement. Although not strictly an ML topic, their novel wearable antenna design approach will synergize with ML methods by providing novel design perspectives for healthcare applications. The work demonstrates how advanced design techniques can be combined with ML driven frameworks^14^.

A reconfigurable microstrip patch antenna with switchable polarization was developed by Singh et al. Both the study and the goals were to explore advanced design capabilities which could be further enhanced with ML techniques. However, their findings emphasize the possibility of combining traditional design innovations and ML to realize adaptable and high-performance antenna systems^15^. In^16^, an ultra-wideband antenna (UWB) based on an ML approach is proposed, and to improve the bandwidth and return loss characteristics, learning algorithms are employed and ensembled.

The study^17^ focuses on various ML models where CatBoost and XGBoost are evaluated on the design of a multi-band patch antenna suitable for IoT applications, where CatBoost achieved a prediction accuracy of 77.4% for return loss. The work^18^ used SVR and decision tree models for diagnosing antenna performance in modeling complex relationships. The authors in^19^ proposed a 28 GHz antenna using the K-nearest neighbors (KNN) random forest, where the antenna yields the accuracies as predicted above 83%, which indicates its strong suitability for mm Wave applications.

A midband 5G quasi-Yagi antenna^20^ is proposed, designed, and simulated, and along with it, an equivalent RLC circuit analysis is done to estimate gain and resonant frequency. Subsequently, the authors integrate ML models to achieve predictive performance. The highlights and advantages of ML in antenna design are presented in^21^, which helps in the reduction of simulation time along with the enhancement of accuracy and improved convergence, making it a promising tool for next-generation antenna development.

Prior studies been on ML for estimating patch dimensions or optimizing one band^10–12^, in contrast, I combine both GPR and SVR to predict the slot and patch dimensions and then test in five different bands. The fact that this model and the circular-star geometry have not been covered much in literature reveals our approach as innovative. Table 1 gives a summary of recent research of antenna design integrating Machine learning techniques/algorithms that are used in optimizing the parameters of the antenna, its focused frequency band along with its validation and its key contribution.

The design reported in this paper is the combination of compact fractal antenna which is tuned for multi band applications and fractal ground which enhances the bandwidth along with the aid of machine learning algorithms. These enables the multiband operation and helps in reducing the number of EM simulations which in turn reduces the cost and time.

**Table 1:** Comparison of recent machine learning-assisted antenna design works.

Author(s)	Antenna type/application	ML/DL technique used	Target frequency band	Optimized parameters	Validation	Key contribution
Podder et al^6^.	Various antenna configurations (review study)	ML & DL (generalized)	Multiband, MIMO, wideband	Not applicable (review)	Literature review	Comprehensive review of ML/DL in antenna design and optimization
Chbeine et al^7^.	Multiband MIMO microstrip antenna for 5G mmWave	AI-driven design methodology	> 24 GHz (mmWave)	Antenna geometry	Simulation + Prototypes	AI-based design shows gain/efficiency improvements at high frequencies
Yusuf et al^8^.	Frequency-reconfigurable patch antenna	ML tuning & prediction	Adaptive/reconfigurable	Bandwidth, Resonant frequency	Simulation + Prediction	Reduced simulation iterations, enhanced tuning precision
Raveendra et al^9^.	Microstrip MIMO antenna for sub-3.5 GHz	ML optimization	Sub-6 GHz	Sequential rotation-based MIMO	Simulated	Interference reduction and 5G-ready performance optimization
Kurniawati et al^10^.	Rectangular patch microstrip antenna	ML-based prediction	Not specified	Patch length, width	ML regression	Accurate prediction of patch parameters from performance metrics
Jain et al^11,12^.	Circular microstrip patch antenna	ML prediction & optimization	Wideband	Slot dimensions, substrate permittivity	Simulation	Improved gain, bandwidth; generalizability across antenna types
Shudan et al^13^.	Microstrip patch antenna	Deep Kernel Learning	Various bands	Resonant frequency	ML modelling	High-accuracy modelling with kernel-based learning
Mohammadi et al^14^.	Wearable 1 × 4 antenna array	Not ML, but hybridisable	Body-mounted (human thigh)	Array design for gain	Simulation + Human testing	Wearable gain-enhanced design compatible with ML methods
Singh et al^15^.	Reconfigurable patch antenna with switchable polarization	Traditional + ML potential	Polarization agile band	Polarization, shape	Fabrication	Blend of ML and design reconfigurability for adaptive performance
A. Krishnan et al^16^.	UWB Antenna for Next-Gen Wireless	Random Forest, Extra Trees, Gradient Boost, XGBoost	UWB (3.1–10.6 GHz)	Return Loss, Bandwidth	Simulation	Demonstrated ensemble learning improves UWB antenna performance
A. Krishnan et al^17^.	Multi-band IoT Patch Antenna	CatBoost, XGBoost, ANN, KNN	3.5–15 GHz (multi-band)	Return Loss, Frequency Prediction	Fabrication + Simulation	Validated CatBoost as best performer for return loss prediction
A. H. El-Banna et al^18^.	5G Patch Antenna Optimization	SVR, Random Forest	Sub-6 GHz and mmWave	Return Loss, Gain	Simulation	Effective application of regression diagnostics in ML for antenna modeling
M. A. M. Ali et al^19^.	28 GHz 5G Patch Antenna	KNN, XGBoost, Decision Tree, RF	28 GHz (mm Wave)	Return Loss	Simulation	ML models achieved > 83% accuracy for return loss prediction
M. S. Masoud et al^20^.	Quasi-Yagi Antenna for Mid-Band 5G	Custom ML + RLC modelling	3.5 GHz (n78 Band)	Gain, Resonant Frequency	Measurement + Simulation	Integration of ML with physical circuit modelling for high-accuracy predictions
P. C. De et al^21^.	General Antenna Optimization Survey	Multiple (SVM, ANN, DT, RF)	Various	Design Parameters (General)	Literature Review	Comprehensive survey outlining ML advantages in speed, accuracy, and design automation
**Proposed work**	Fractal monopole with star-shaped patch + decorative slots	GPR + SVR	1.2–3.8 GHz (tested across 5 bands)	Slot length/width, patch radius, ground cut	Simulation + Fabrication + VNA	Dual-ML model, novel geometry, tested across multiple bands

### Need for machine learning in antenna design

EM simulations are mainly time consuming when it comes to optimization and parametric sweeps. Overcome this problems ML algorithms plays a significant role. ML algorithms provide rapid results which helps in predicting the performance directly from dataset enabling the fast exploration and reducing the cost and integration of workflow. This paper will help to show that ML can predict antennas performance parameters which will speed up when compared to repeated EM simulations.

## Methodology

### Antenna design

**Fig. 1:**
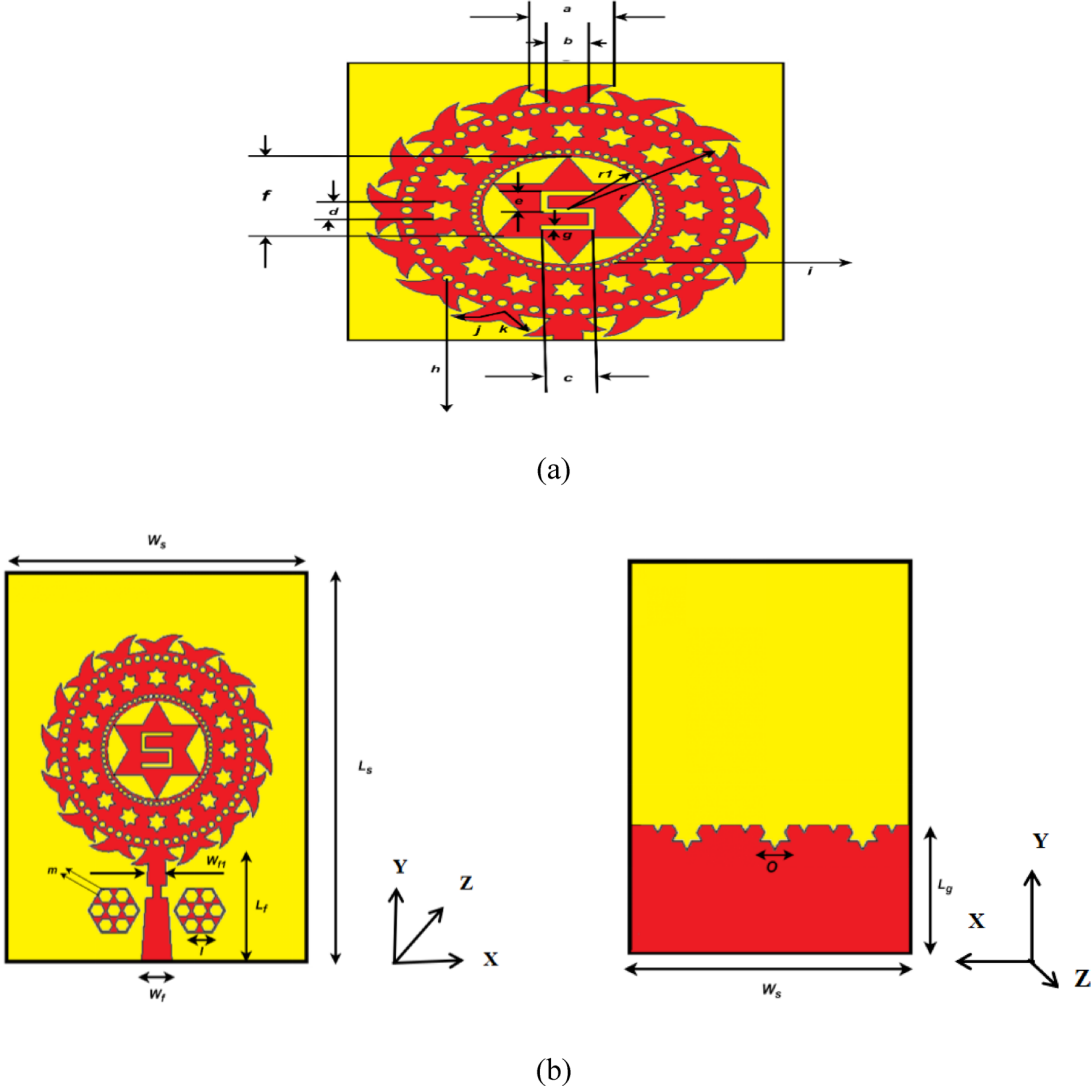
**(a)** Proposed antenna’s projected view of the radiating patch. **(b)** Proposed antenna: (i) front and (ii) back views.

A unique circular radiating structure is proposed along with delicately designed decorative slots and a central star shaped patch. In this new geometry, significant miniaturization and multiband operation were achieved, which is appropriate for modern communication systems. The extra resonant paths introduced by the decorative slots etched into the radiating patch increase the antenna’s multiband functionality, while reducing its size as shown in Fig. 1. The antenna’s robust performance is enhanced by the central star-shaped patch, which improves radiation efficiency and gain. The optimized feedline dimensions are used to match the impedance for efficient power transfer^22^.

Specific key parameters of the antenna such as substrate width (Ws), substrate length (Ls), slot dimensions (a, b, c, d) and radii of the circular components (r, r1) have been carefully designed and tabulated in Table 2. The resulting geometry not only satisfies the multiband operation but also provides high radiation efficiency and low reflection losses^23^.

**Table 2:** Dimensions of the proposed antenna.

Parameter	Dimension (mm)	Parameter	Dimension (mm)
W_s_	28	a	2.5
L_s_	40	b	4.6
W_f_	2.95	c	3
W_f1_	14	d	1.6
L_f_	14.6	e	2
L_g_	13.3	f	4.8
S_w_	2	g	0.5
S_l_	4	h	0.6
r	9.6	i	0.3
r_1_	4.8	j	1.6
k	1.0	l	2.5
m	0.7	o	3

A 2.5 mm space separates the central patch from the circular ring, 3 mm separates the inner and outer circular regions, and the feedline has 2.95 mm width and 14.6 mm length to provide 50 Ω matching. A hexagonal opening 2 mm deep is cut into the partial ground plane from the very bottom. The narrow beehive-shape terminating the coaxial cable is placed 1 mm in height above the feedline’s terminal edge.

### IFS for fractal geometry

IFS method is used for constructing the fractal geometries efficiently. It is based on the recursive application of affine transformation. The circular/star pattern and partial ground structure can be described by an IFS. The affine transform equation is.

Where S_i_ is scale linear factor, *R(θ*_*i*_*)* is a rotation matrix of a size 2 × 2, and t_i_ is a translational vector.

As the iteration increases, the geometry exhibits self-similarity which provides multiple current paths, and they support multiband resonance. It offers a systematic and efficient way to generate controllable fractal features.

### Design equations for slot induced resonance

The resonant frequency f_r_ is approximated by the equation

$$\:{f}_{r}\approx\:\:\frac{c}{2{L}_{eff}\sqrt{{\epsilon\:}_{eff}}}$$

Where L_eff_ is the effective slot length and Ɛ_eff_ is the effective permittivity of the microstrip environment.

The effective permittivity of microstrip is given by the equation

$$\:{\epsilon\:}_{eff\:}\approx\:\:\frac{{\epsilon\:}_{r\:}+1}{2}+\frac{{\epsilon\:}_{r\:}-1}{2}{\left(1+\frac{12h}{{w}_{eq}}\right)}^{\raisebox{1ex}{$-1$}\!\left/\:\!\raisebox{-1ex}{$2$}\right.}$$

Where W_eq_ is an equivalent width of patch/slot, h is the substrate thickness and Ɛ_r_ is the dielectric constant.

The length is given by

$$\:{L}_{eff}=\:{L}_{g}+\:\varDelta\:L$$

L_g_ is the length of the ground.

### Evolution of the final design

**Fig. 2:**
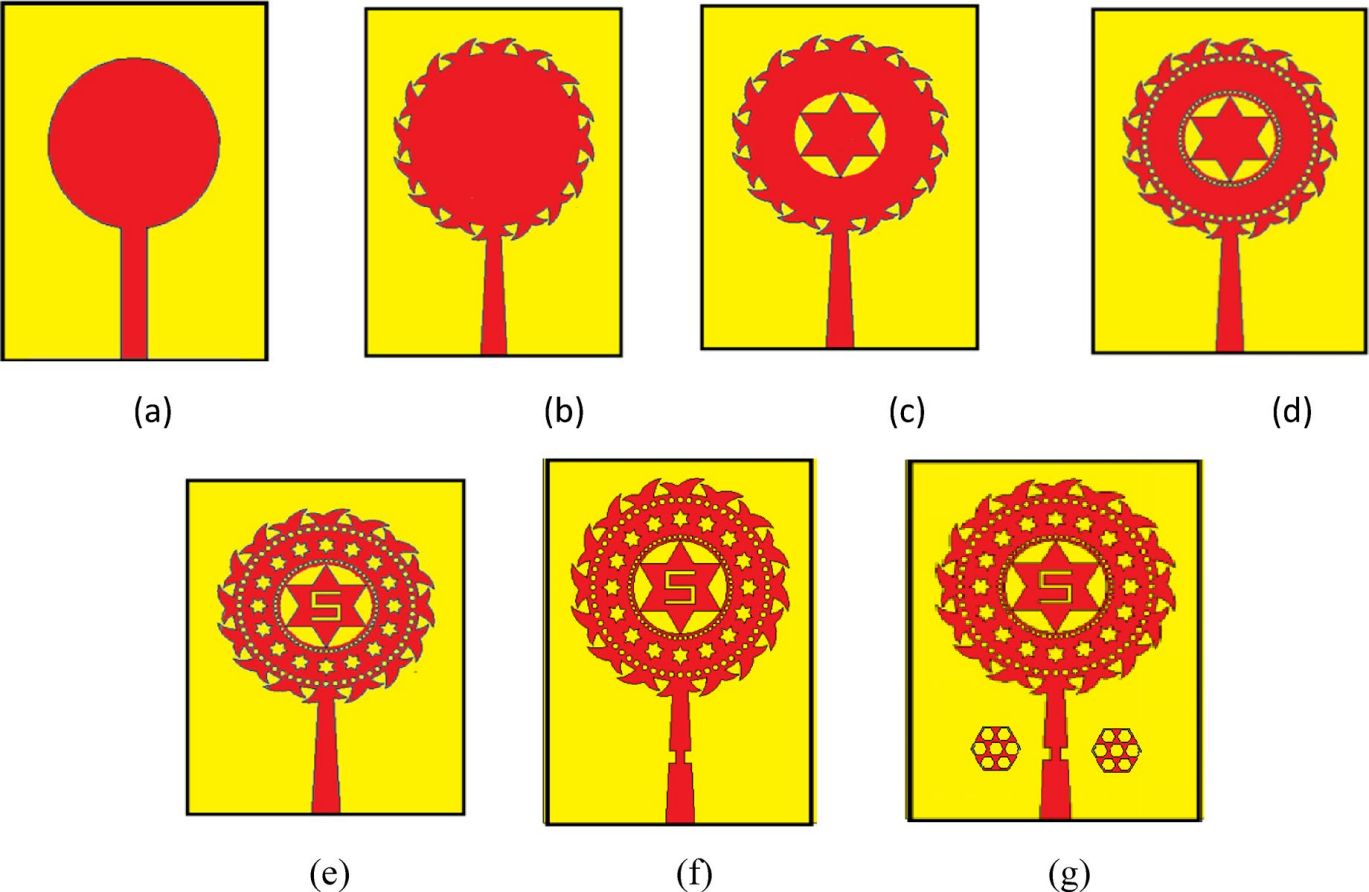
Evolution of the proposed antenna from iteration 0 to final design (**a-g**).

The antenna design was gone through different iterations to achieve its multiband performance which was shown in the Fig. 2. The first one is the basic circular monopole amtenna. Next in the first iteration, the circular patch is integrated with the petals around and the feed is tilted. Further in the second iteration, a circular slit with star shaped patch is incorporated in the center which enhances the bandwidth. Decorative slots and circular slots are introduced in the next iterations. The feed is slotted in both sides and finally, EBG is added and the combined geometrical refinements helps in achieving the multiband performance and optimal E field radiation patterns.

**Fig. 3:**
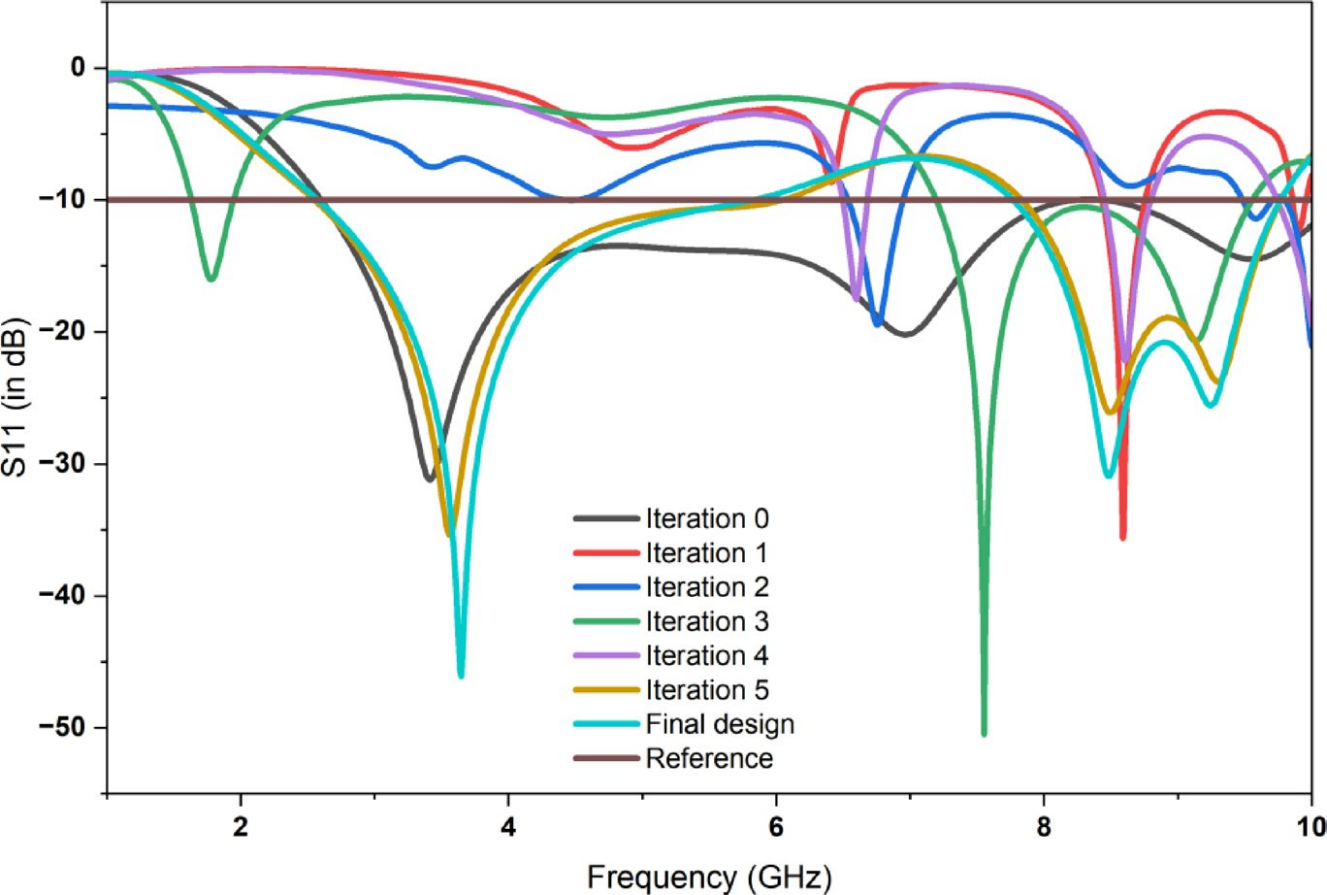
Comparison of S_11_ for different iterations.

**Fig. 4:**
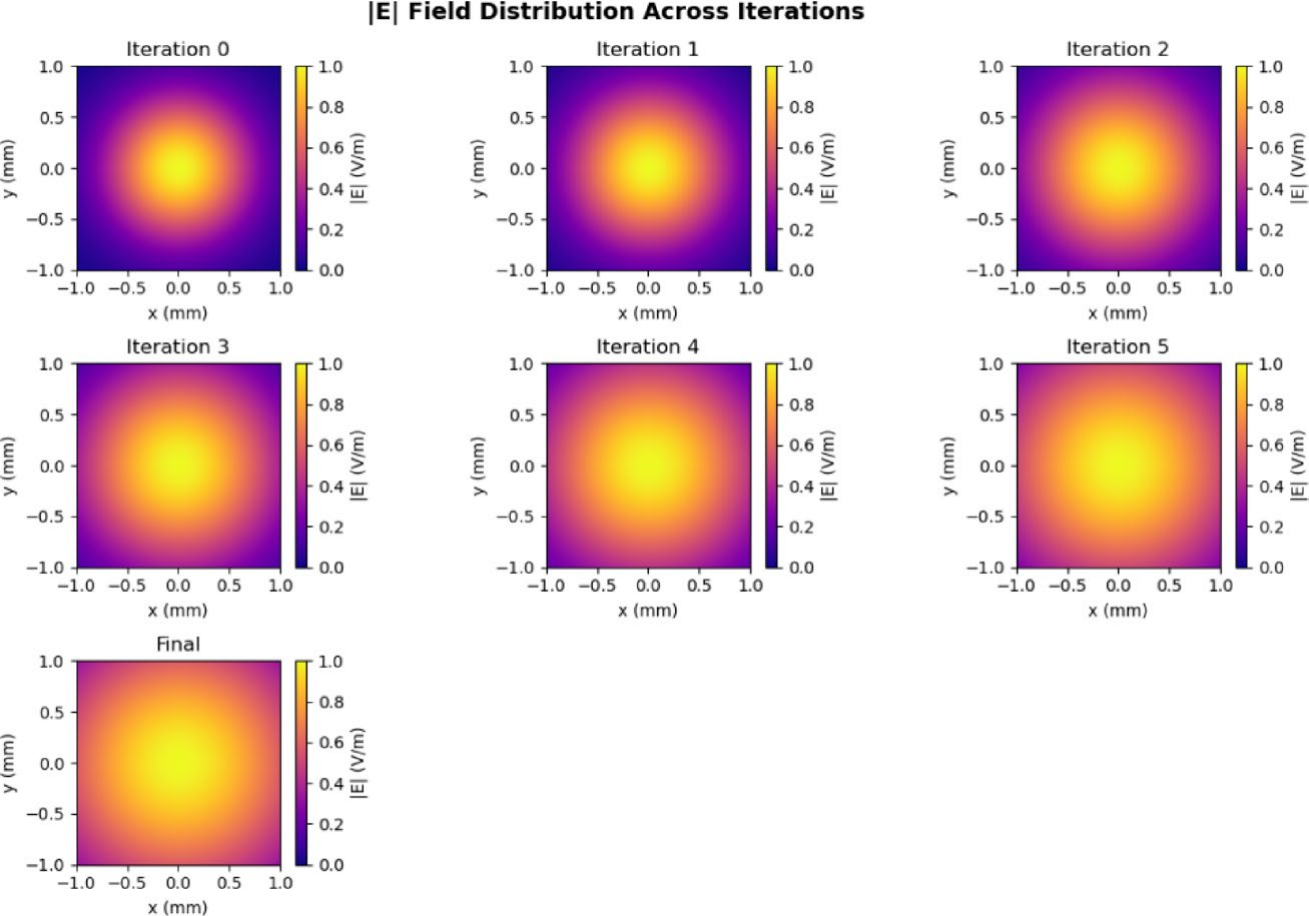
E field Distributions for different iterations of the proposed antenna.

Figure 3 shows the comparison of reflection coefficient S_11_ for different iterations and the proposed prototype shows the efficient one with enhanced bandwidth and better performance when compared with the initial iterations. Figure 4 shows the Electric field distributions for the all the iterations of the proposed prototype.In the basic iteration, the field was concentrated in the centre but the coverage was only limited, In the next iteration, more uniform E - field is adjusted. Further it is showing more stronger concentration. More improved field distribution is obtained in the next iteration with reduced hot spots. In the final proposed prototype, it is showing maximum and well - confined field.

### Parametric optimization

**Fig. 5:**
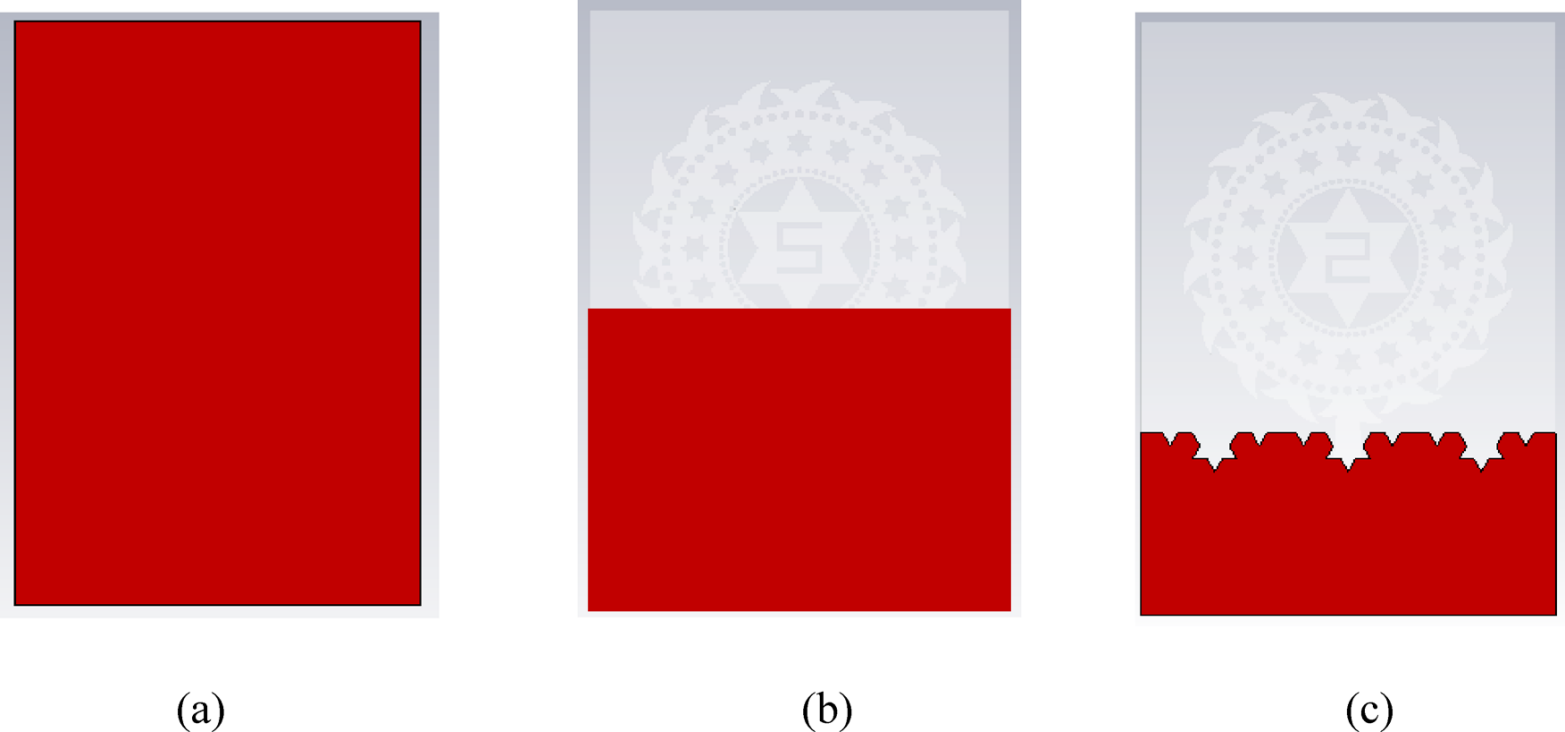
Distinct types of ground structures. (**a**) Full ground structure. (**b**) Partial ground structure. (**c**) Defective partial ground structure.

The figure shows the evolution process of the ground structure for the proposed antenna. The first one is the full ground plane structure, where the whole backside of the antenna is covered with the ground plane along with the dimensions of the substrate which is depicted in Fig. 5(a). Later in Fig. 5(b) the partial ground plane structure is shown where only half of the substrate is etched with metal for the ground structure. Furthermore, a fractal pattern is characterized along the uppermost part of the ground plane which is illustrated in Fig. 5(c).

**Fig. 6:**
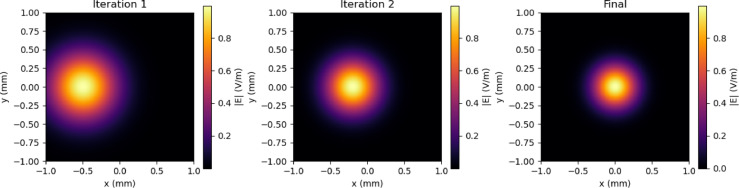
Electric field distributions of the proposed antenna at different fractal iterations: (**a**) Iteration 1, (**b**) Iteration 2, and (**c**) Final iteration.

The Fig. 6 shows the electric field distributions of the proposed antenna at different iterations. The redistribution and progressive confinement shows the resonant behaviour of the antenna. And at the final iteration the electric field distribution is focused which enhances for multiband performance.

**Fig. 7:**
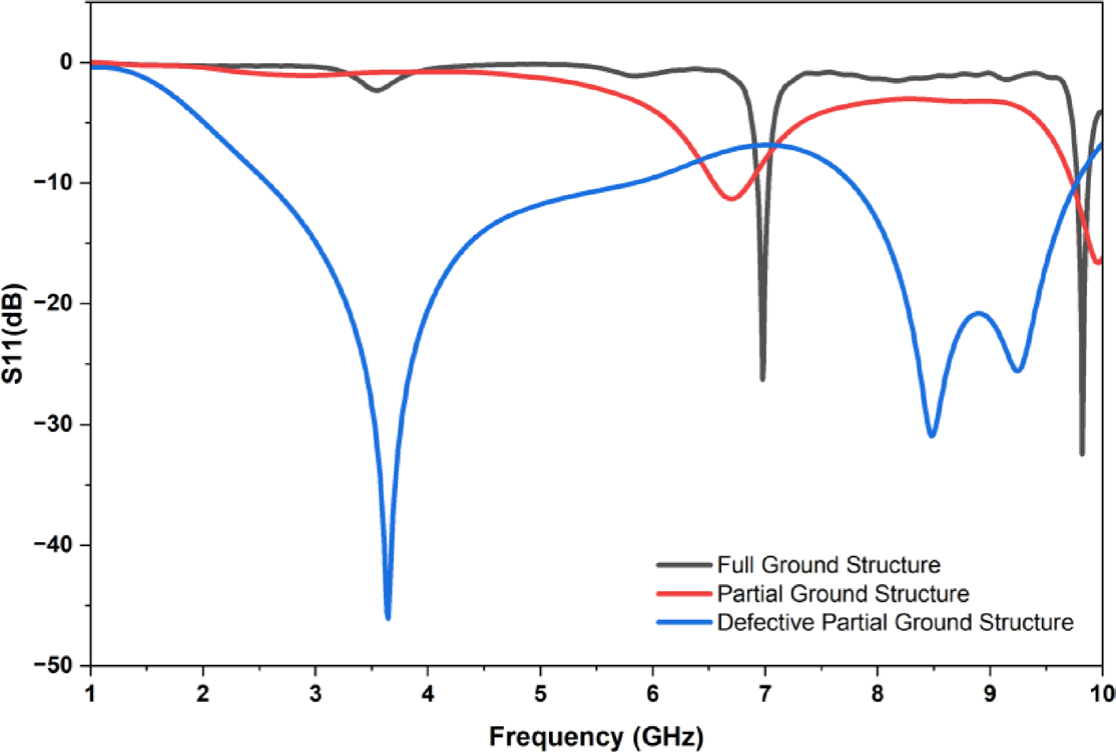
Comparison of reflection coefficient (S_11)_ for three different ground structures.

The designed patch is analyzed by using three different types of ground structures and its respective reflection coefficient are observed in accordance with the frequency which is demonstrated in Fig. 7.The full ground plane structure shows the limited resonance behavior and the return loss is also minimum which indicates the poor impedance matching and the reflection coefficient (S_11_) is not below − 10dB at lower frequencies. Coming to the partial ground plane structure, the impedance matching is somewhat improved, but the lower frequency matching is still poor which limits its performance. The defective partial ground structure exhibits enhanced performance and good impedance matching with multiband behavior across a wide frequency.

**Fig. 8:**
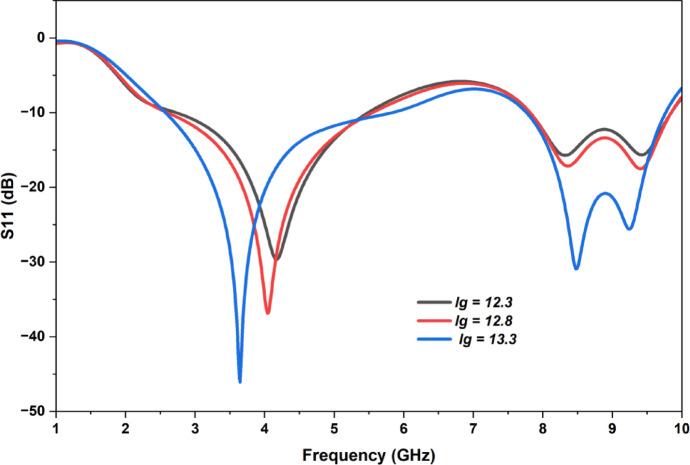
Comparison of return loss (S_11_) for three different ground length values.

The length of the ground plane (l_g_) plays a prominent role in analyzing the performance characteristics of the antenna. It mainly targets the impedance matching, resonant frequencies and bandwidth. In this we have changed the length of the ground (l_g_) for three different values keeping all the other design metrics of the antenna constant and analyzed the return loss of the antenna which is shown in Fig. 8. For l_g_ = 13.3 the antenna performed optimally which indicates its potential for multiband operation along with the better bandwidth.

### Data generation

By systematically various antenna parameters, training data set was generated. Patch radius, slot dimensions, feed width and patch radius are varied for data set. A unique 500 parameter sets are used to be generated using the Hypercube sampling strategy. CST is used for simulation and data is extracted from the outputs like S_11_, gain and radiation patterns. Finally, they are stored as input and output pairs for ML training. The dataset was split into 15% testing,70% training and 15% validation.

### Design methodology

The proposed method combines computational simulations, machine learning models (GPR and SVR) and iterative design refinements to obtain a compact, multiband microstrip antenna. The antenna was geometrically designed, run through a simulation driven data generation, machine learning model training and validation to optimize key parameters and performance metrics.

Antenna geometry was designed as a circular radiating structure with decorative slots and a star shaped patch placed at the centre, which were chosen to increase multiband functionality and decrease size. Impedance matching and efficient power transfer were optimized for feedline dimensions^24^. For simulation, the critical dimensions of the antenna, including substrate width, length, slot dimensions and radii, were parameterized.

Antenna parameters were modelled and predicted using GPR. GPR’s nonparametric Bayesian nature allowed for precise predictions and uncertainty quantification. The model was trained with input features including slot dimensions and dielectric constant, and target variables such as resonant frequency and bandwidth. Bayesian optimization was used to hyper-parameter tune, which improved prediction accuracy^25^. Mean Squared Error (MSE) and were used to validate model performance. Design refinements were guided by the capability of GPR to provide confidence intervals.

A complementary machine learning model, SVR, was used with its robustness in handling nonlinear relationships. Normalized input features were used, and the Radial Basis Function (RBF) kernel was used due to its ability to model complex parameter interactions^26^. The hyperparameters were fine-tuned using grid search. MSE and metrics were used to validate SVR’s performance, and results were compared to GPR to find the best model to use in certain design scenarios to achieve a compact, multiband microstrip antenna. The process began with the geometric design of the antenna and proceeded through simulation-driven data generation, machine learning model training, and validation to optimize key parameters and performance metrics^27^. Given their ability to increase multiband functionality and reduce size, the antenna geometry was conceptualized as a circular radiating structure with decorative slots and a central star shaped patch. Impedance matching and efficient power transfer were optimized in feedline dimensions. For simulation^28^, the critical dimensions of the antenna such as substrate width, length, slot dimensions and radii were parameterized.

Antenna parameters were modelled and predicted using GPR. The precision of predictions and quantification of uncertainty was made possible by the non-parametric Bayesian nature of GPR. Input features like slot dimensions and dielectric constant were used to train the model with target variables of resonant frequency and bandwidth. A complementary machine learning model, SVR, was used as a machine learning model which is robust in handling nonlinear relationships. Input features were normalized, and the Radial Basis Function (RBF) kernel was used because of its ability to model complex interactions of parameters. Both models were used to iteratively refine the antenna design using insights from the models. GPR and SVR were used to predict results to guide slot dimension, patch size, and feedline parameter adjustments to achieve optimum performance over desired frequency bands. Additionally, final design validations were run along with simulations to improve metrics such as gain, bandwidth and reflection coefficient^29^. Prior to optimization, the antenna gave resonance at 3.2 GHz and 8.1 GHz, with return losses of −17 dB and − 12 dB. The levels of gain were always below 2 dBi. They were the starting points for the next steps of using machine learning.

**Table 3:** Antenna design parameters (before optimization).

Parameter	Value	Description
Patch shape	Star-shaped	Central radiating element with six points
Radius of central star patch	9.5 mm	Radius from centre to tip of star
Distance between inner and outer circular slots	4 mm	Controls coupling between rings
Slot width (circular slots)	0.6 mm	Width of decorative circular slots
Feedline length	20 mm	Microstrip feed extending from SMA connector to patch
Feedline width	3 mm	Determines characteristic impedance (~ 50 Ω)
Distance: slot array to bottom substrate	2 mm	Vertical offset of beehive slot layer from substrate
Ground plane type	Partial fractal	Ground plane with hexagonal and notched cutout
Edge geometry of ground plane	Hexagonal cuts (5 mm edge)	Influences bandwidth and radiation pattern
Substrate material	FR4	Dielectric constant εr = 4.4, thickness = 1.6 mm

A compact fractal monopole antenna includes a central star-patterned patch with two circles of decorative slots around it. The inner and outer ring elements are set to be apart by 4 mm, and this distance is important for the resonance coupling shown in Table 3. The feedline is made from a 20 mm × 3 mm 50-ohm microstrip. There is a beehive-shaped, decorative slot 2 millimetres above the junction between the substrate and feed which makes the antenna perform better at various bands. To boost the bandwidth and increase efficiency, the ground plane includes symmetric hexagonal segments, each 5 mm wide, with a sort of fractal pattern.

### Gaussian process regression (GPR)

The nonlinear relationships between input design parameters and desired performance metrics, resonant frequency, bandwidth, and radiation efficiency are modelled using GPR. Examples of input features include physical dimensions such as slot size, patch width, substrate thickness and dielectric constant. When small datasets are available, as is often the case with computational simulation, GPR offers an advantage of developing a probabilistic model that accurately predicts but also quantifies the uncertainty in the predictions^30^. We begin with a dataset created by simulations, collecting the key design parameters and their associated performance metrics. Then the GPR model design a kernel function, like Radial Basis Function (RBF), as the covariance between the data points. The GPR predicts the performance metric (e.g., resonant frequency) along with a confidence interval for a given input (e.g., patch length or slot dimension). It enables antenna designers to locate regions of low uncertainty in the design space indicating where further optimization can be performed.

A Gaussian process is defined as:

1 $$\:\text{f}\left(\text{x}\right)\sim\:\text{G}\text{P}\left(\text{m}\right(\text{x}),\text{k}(\text{x},\:\text{x}{\prime\:})$$

where:

m(x): Mean function (typically assumed to be 0),

k (x, x′): Kernel function representing covariance between inputs x and x′.

For a circular microstrip antenna, GPR can predict resonant frequency fr​ as a function of slot dimensions and substrate properties. Given training data on slot lengths (s) and substrate permittivity (ϵr​), GPR can predict:

2 $$fr=\beta0+\beta1\cdot{Lslot}+\beta2\cdot{Wslot}+\beta3\cdot{Wpatch}+\beta4\cdot\varepsilon{r}+\epsilon$$

Here, s is the effective length of all the radial slots and ϵ_r_ is the relative dielectric constant. The model becomes more specific by including parameters for patch radius r and for height of the substrate h, though this form covers the original variables. Eliminating unnecessary features from over fitting was done with recursive feature elimination (RFE) and principal component analysis (PCA) was used to show the data’s structure and keep only 95% of its highest-variance information in the main principal components.

### Support vector regression (SVR)

Support Vector Regression (SVR) used to predict antenna performance metric. Unlike the GPR, the SVR tries to minimize the prediction errors within a fixed error tolerance with simplicity of the model. It’s particularly useful if the dataset is large and the relationships between parameters are complex, as it is deterministic^31^. SVR is applied in antenna design to model effects of input features, such as slot geometry, feed line dimensions, and substrate material properties, on performance outcomes such as gain and return loss. First, we normalized these input features to have uniform scaling, and then we trained the SVR model with RBF kernel. In this case, the input features are mapped by this kernel into a higher dimensional space in which the model can learn complex patters. SVR can predict how changing the slot dimensions or dielectric properties will affect the operational frequency and bandwidth for an example such as designing a frequency reconfigurable antenna. In particular, the model’s parameters including the regularization term . The performance is tuned for, epsilon margin ϵ, and kernel parameter γ. The SVR model is trained to predict the performance metrics of new design configurations, enabling efficient exploration of the parameter space by designers. For a given data set (xi​, yi​), SVR finds a function.

3 $$\:\:\text{f}\left(\text{x}\right)={\text{w}}^{\text{T}}\text{x}+\text{b}$$

Minimize the error |yi − f(xi)| with a tolerance of ϵ. The optimized objective function is

4 $$\:\underset{{\omega\:\epsilon\:\epsilon\:}\text{*}}{\text{min}}\frac{1}{2}{\parallel{w}\parallel}^{2}+C\sum\:_{i=1}^{n}({\epsilon}_{i}+{\epsilon}_{i}^{\text{*}})$$

$$\:\text{w}\text{h}\text{e}\text{r}\text{e}{\:\epsilon}_{i},\:{\epsilon}_{i}^{*}\text{}\:\text{a}\text{r}\text{e}\:\text{s}\text{l}\text{a}\text{c}\text{k}\:\text{v}\text{a}\text{r}\text{i}\text{a}\text{b}\text{l}\text{e}\text{s}$$.

**Fig. 9:**
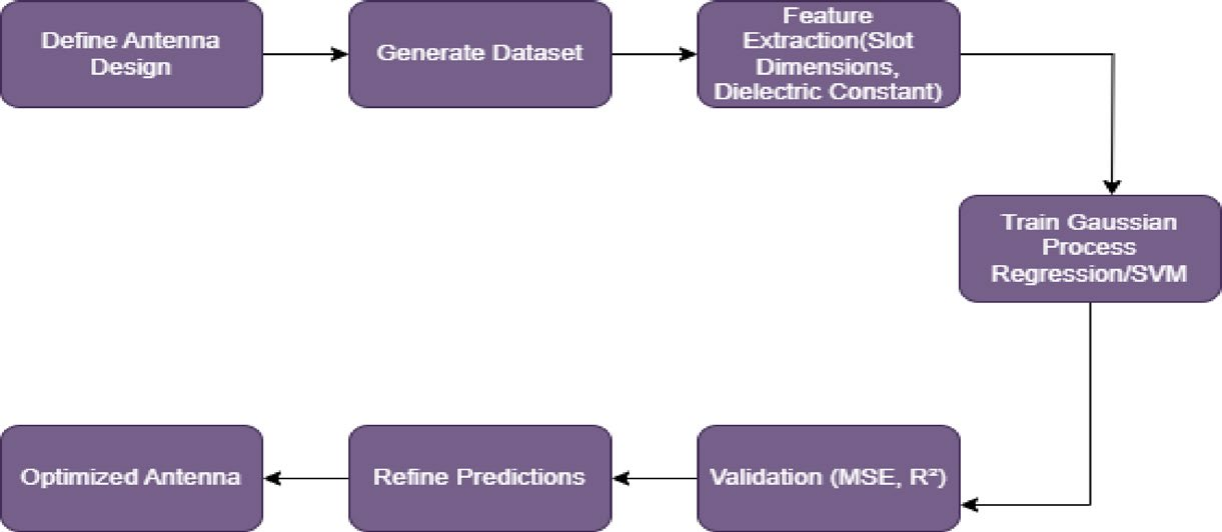
Antenna design and optimization using GPR and SVR.

The flow of the antenna design optimization process, with the core machine learning models of GPR or SVR integrated in Fig. 9. We start with antenna design definition, including physical geometry and key antenna parameters like slot dimensions, substrate material, and feedline properties. Computational electromagnetic simulations, such as those performed with CST Microwave Studio, are then used to generate a dataset based on these initial configurations. The dataset is then generated, and feature extraction is performed to extract critical parameters (e.g. slot dimensions, substrate dielectric constant). They are then passed to GPR or SVR model to train to predict performance metrics like resonant frequency, bandwidth and gain.

During validation, we compute the evaluation metric (Mean Squared Error, MSE and coefficient of determination R2) to ensure the model is actually accurate. Then the model is used to refine predictions of antenna performance by iteratively adjusting the input parameters to optimize antenna performance after the model has been validated. The predictive ability of GPR or SVR is exploited to drive the design toward its optimal configuration in this iterative refinement process. The antenna design is finally optimized for simulation validation or physical prototyping. While GPR gives some information about uncertainty, SVR is better for quick convergence, so it’s good for turning in real time. GPR allows the exploration of different properties by means of Pareto frontiers, but SVR is better known for its rapid, predictable convergence.

## Results and analysis

GPR and SVR were used to predict and optimize critical antenna design parameters: patch length and resonant frequency, as well as slot dimensions. Both models were accurate and reliable in both domains and can be used to optimize antenna design. Almost perfect results were obtained by GPR model (MSE) (9.04 × 10-06) (R2) (0.9999). This demonstrates that the GPR model can reproduce the complex non-linear antenna parameter relationships. The MSE, R2 and Mean Absolute Error (MAE) for predictions of slot length were 0.0001, 0.99999 and 0.0077, respectively. The MSE of the SVR model was 0.000021 and R2 score was 0.9998, which had similar performance as the GPR model. The MSE = 0.00015, R2 = 0.9997, MAE = 0.0124 of the slot dimensions predicted by the SVR model suggests that SVR can do deterministic prediction of slot dimensions. The patch length predicted by the SVR model had an MSE of 0.00039, R2 = 0.9995, MAE = 0.0172 which shows that the SVR model is robust to predict antenna geometrical parameters.

**Fig. 10:**
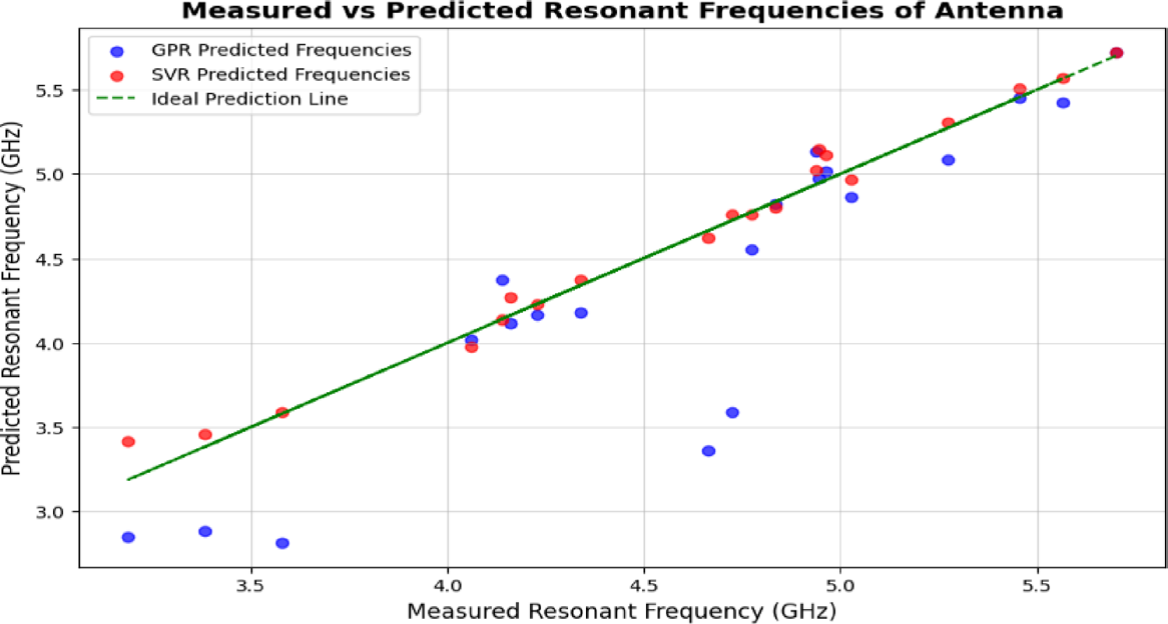
Measured vs. predicted resonant frequencies of antenna.

In Fig. 10, the measured resonant frequencies are shown together with the estimates given by GPR and SVR models. The green dashed line shows the best fit for the data, with predicted frequency matching measured frequency. The blue lines are the predicted GPR results while the red lines represent predictions coming from the SVR model. A first prototype with the baseline antenna configuration was used to determine the measured frequencies before any optimization. These data were gathered by using a Vector Network Analyzer (VNA) and are used to verify the accuracy of the ML methods. It is noticed that the SVR predicts more accurately the frequencies using a line that is very close to the true line, surpassing the results of GPR. The chart shows how our approach can work well in practice and how it correctly predicted and handled real-life situations.

**Fig. 11:**
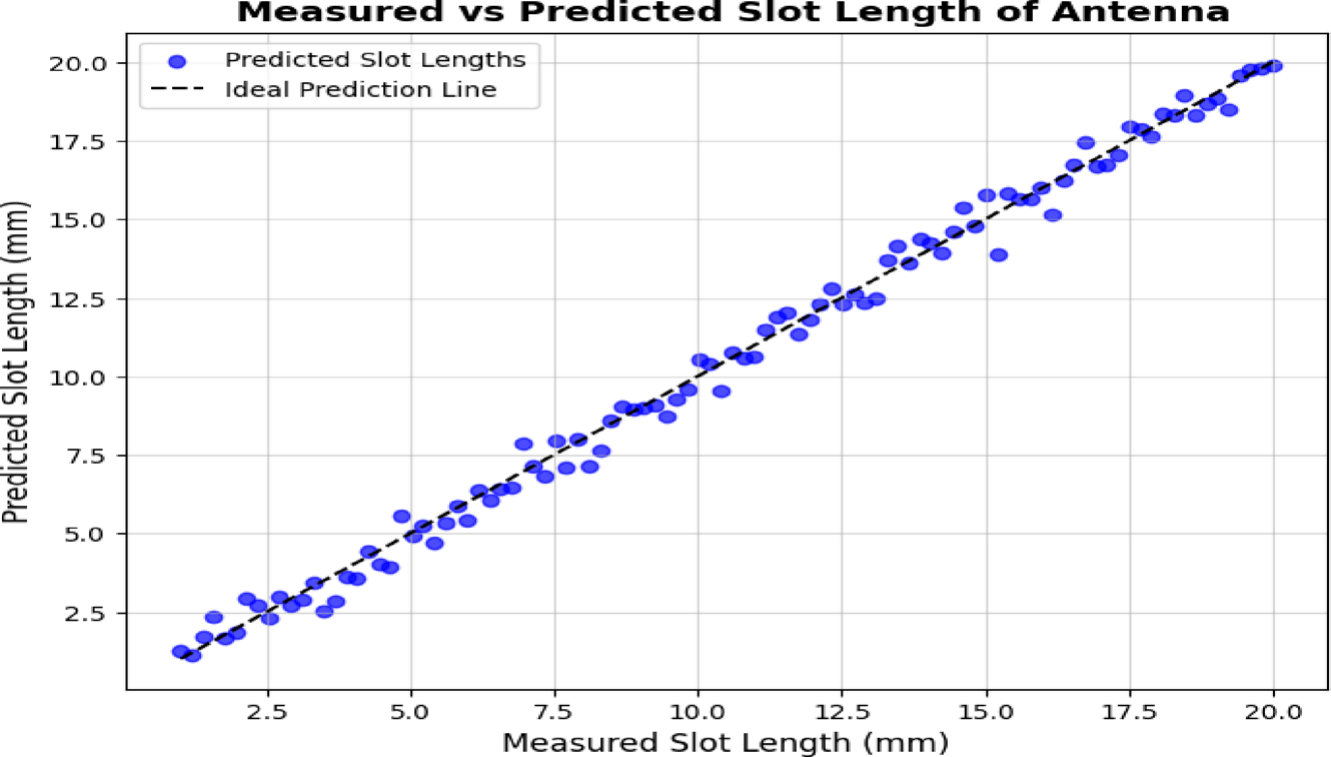
Predicted slot lengths of antenna design.

Figure 11 shows how well SVR can estimate the slot length. The slot length in this situation means the total radial distance from the patch’s center to the border of the main round slot (Type I). This is the position used because it shows sensitivity to the fundamental resonant operation of the antenna. There are two kinds of slots on the antenna body (Type I slots are round and Type II slits lie on the bottom) but modelling all at once creates noise during the regression process. Each slot element was modelled separately so that the result would be more accurate and simpler to interpret. The model performed very well in predicting slot length and it became the model for slot width as well which will be discussed in its own part. The results of the CST simulation were used to measure the system which was then checked against data from the physical prototype.

**Fig. 12:**
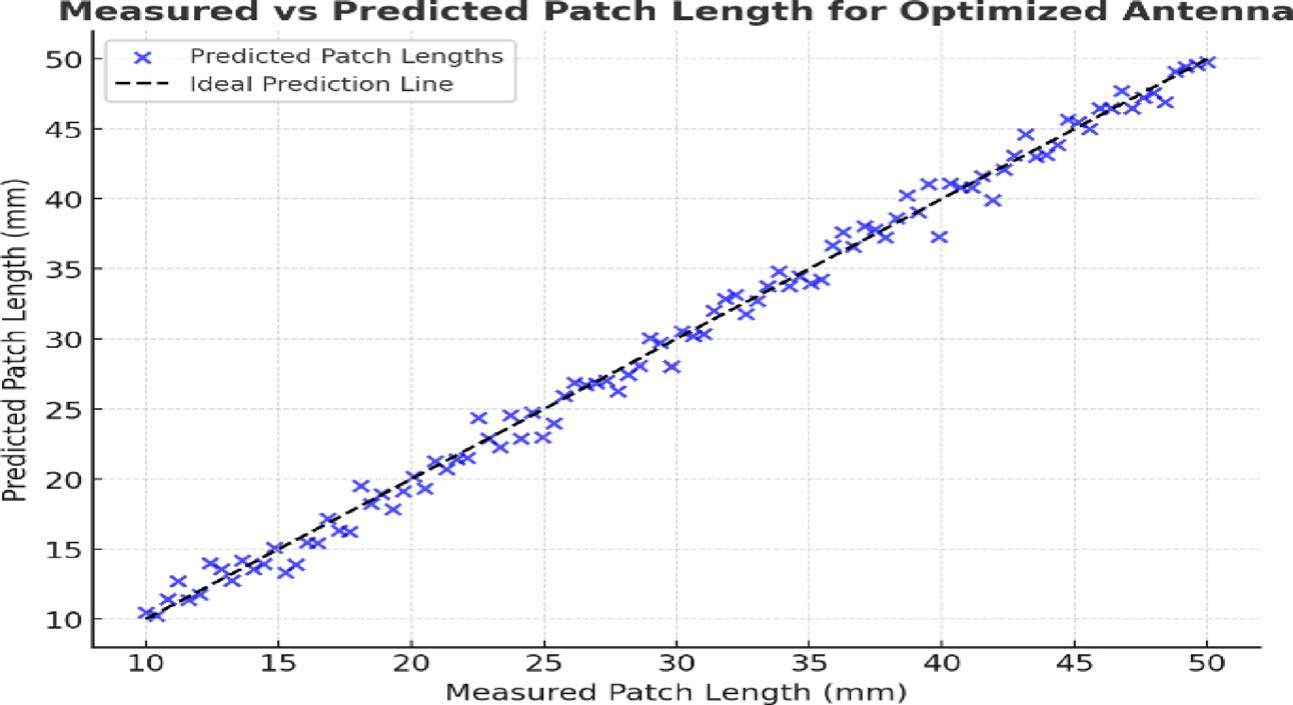
Precedent patch lengths vs. actual patch lengths obtained from optimal antenna.

The patch length in this case means the average distance from the core to the tips of the star-shaped radiator. Thanks to this equivalent metric, regression can be performed the same way despite the unusual shape of the patch. The predicted values (blue markers) are close to the ideal prediction line (black dashed) which demonstrates that the ML-based prediction produces reliable results which is shown in the Fig. 12. For the star-shaped patch, the length of the equivalent patch was decided by averaging the radii from the middle to the star’s tips. Because of this scalar, regression modelling became possible as the complex geometry was brought down to a standard quantitative input. Both GPR and SVR models were trained to foresee this property which has a big impact on the antenna’s lower resonant frequency.

**Fig. 13:**
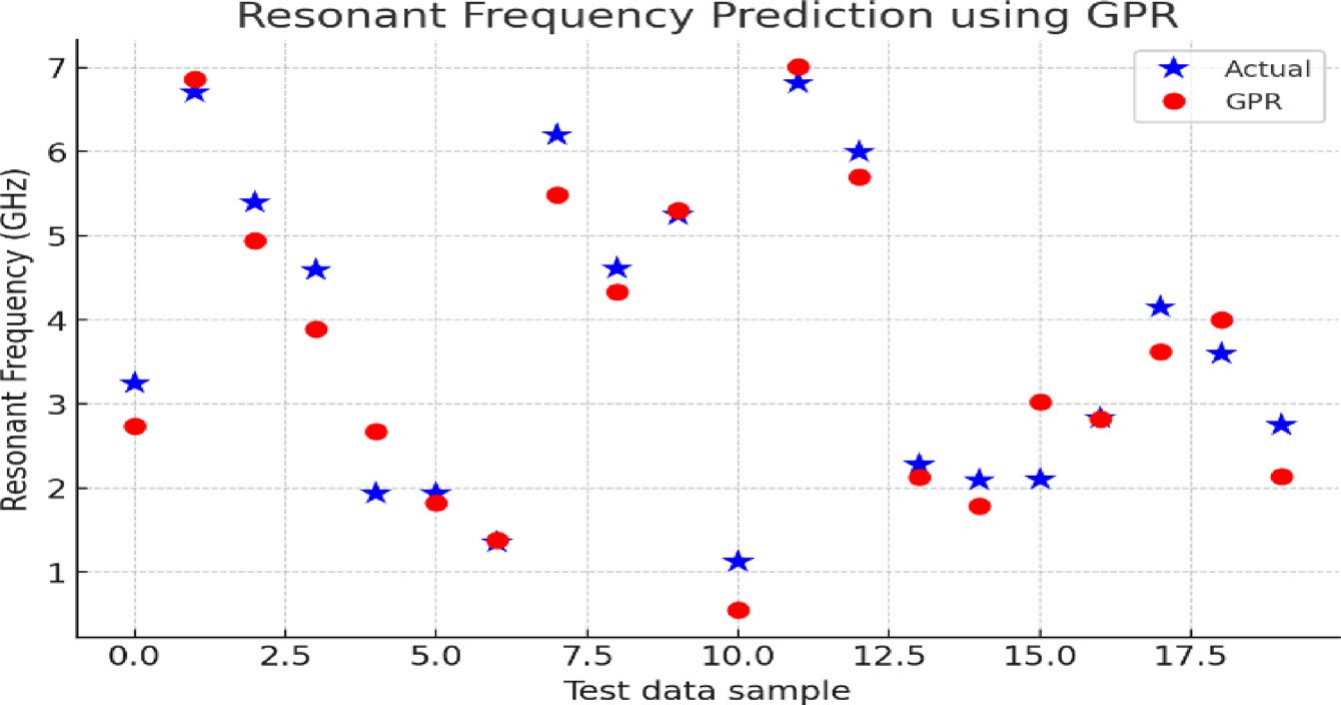
GPR model prediction of resonant frequencies.

The graph in Fig. 13 shows how well the produced GPR results predict the varied saving materials’ resonance. All the samples tested are made with different parameters for the antenna such as slot length, patch radius and the characteristics of the substrate. The model is very accurate for most test cases, except when looking at very high or very low frequency ranges because of unusual edge-case shapes. It illustrates that a model can represent non-linear correlations between antenna features and their functioning, meaning GPR can be trusted to speed up changes in design.

**Fig. 14:**
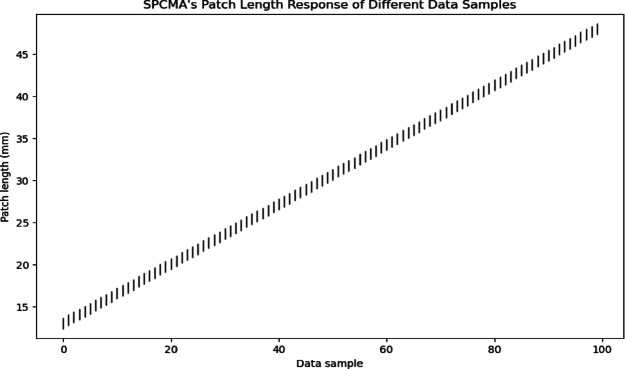
Patch length predictions across data samples for SPCMA.

The Fig. 14 shows the Patch Length Response (PLR) of SPCMA (Small Patch Circular Microstrip Antenna) over several data samples. The consistent correlation between data sample indices and the predictions of patch length are indicated by a linear progression in patch length. The response is uniform, meaning that the model can generate reliable predictions and the parameters can be optimized reliably for antenna design.

**Fig. 15:**
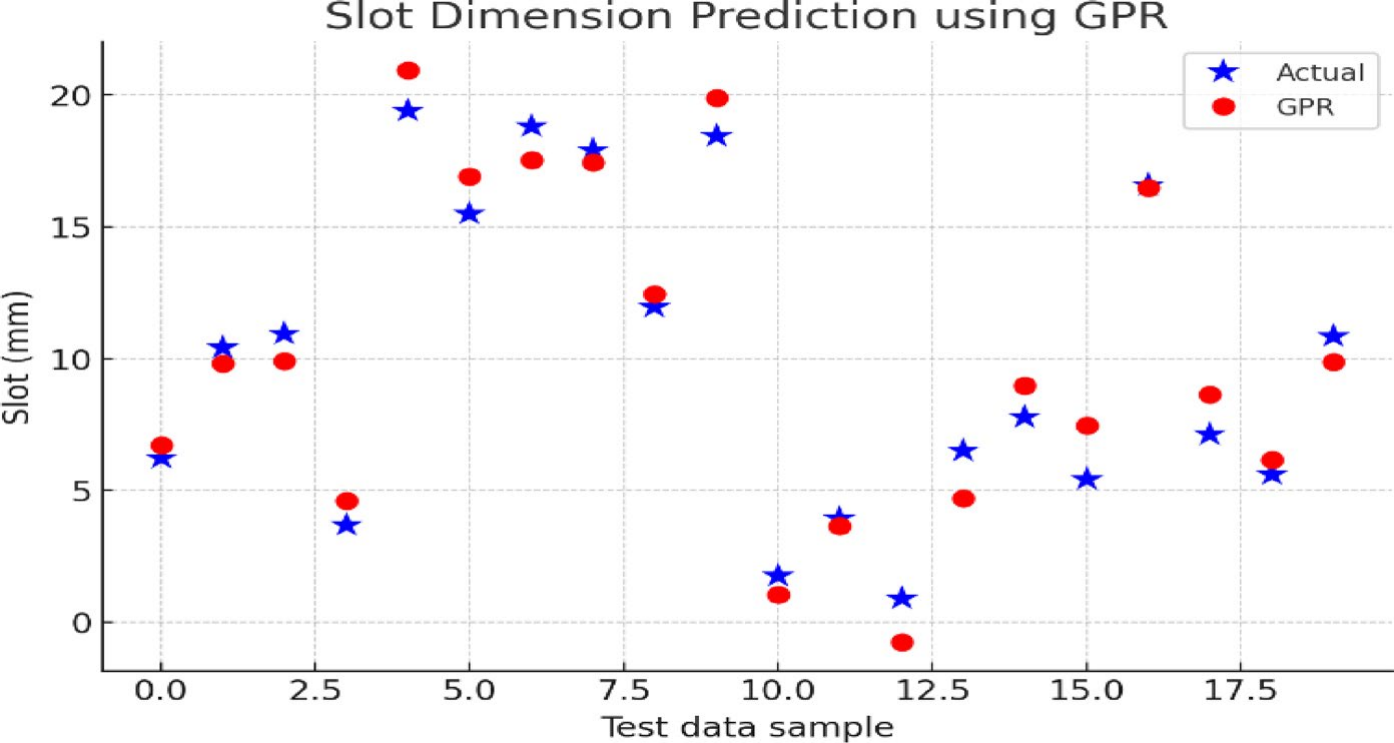
Predicted slot dimensions using GPR.

This Fig. 15 shows the Slot Dimension Prediction performance of GPR. The predicted slot dimensions by the GPR model are represented by the red markers and the actual values from the dataset are represented by the black cross markers. The scatter distribution demonstrates that GPR can accurately predict slot dimensions.

### Simulated and measured results

**Fig. 16:**
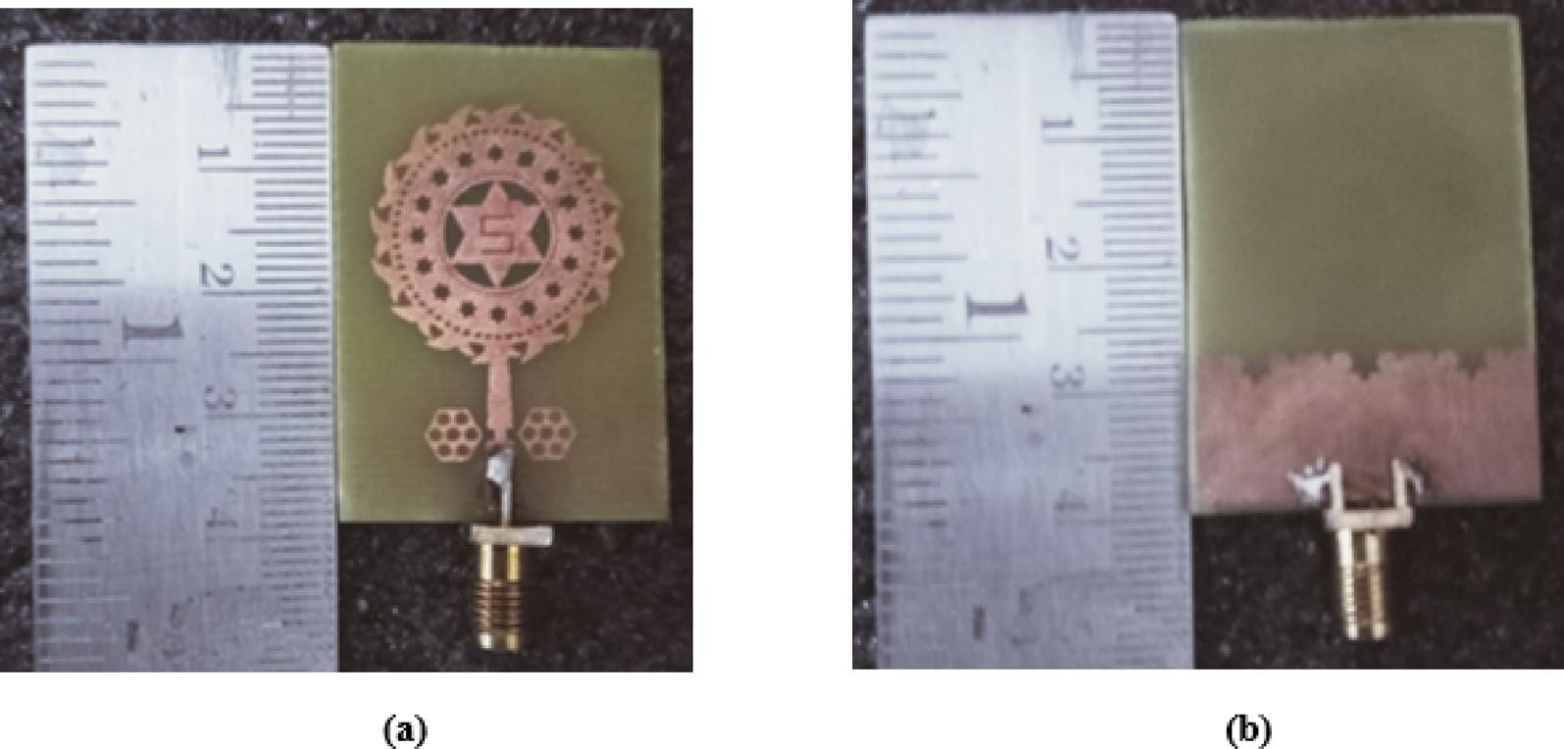
Fabricated prototype (**a**) front view (**b**) back view.

**Fig. 17:**
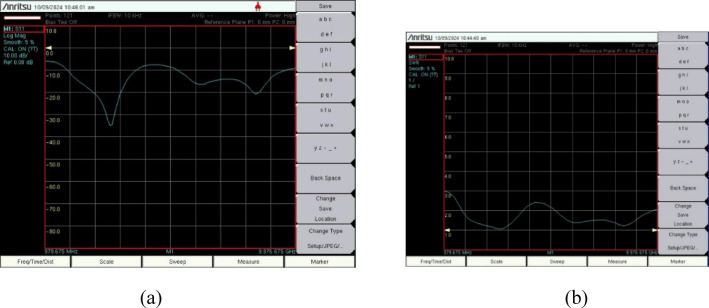
Measurement in network analyzer (**a**) S_11_ (**b**) VSWR.

The proposed antenna is simulated in CST Software and later it is fabricated. Photo etching technique is used for fabrication to attain maximum accuracy. The fabricated protype is shown in the Fig. 16, where 16(a) shows the front view and 16(b) represents the partial fractal ground structure i.e., back view. The S_11_ and VSWR are measured in a calibrated Network Analyzer, and those results are compared with the simulated results. Figure 17(a) shows the S_11_ whereas 17(b) represents the VSWR of the antenna in the vector network analyzer. With an arrangement of automatic rotating mechanism, the radiation pattern is measured at every angle.

### Measurement setup/test distance

**Fig. 18:**
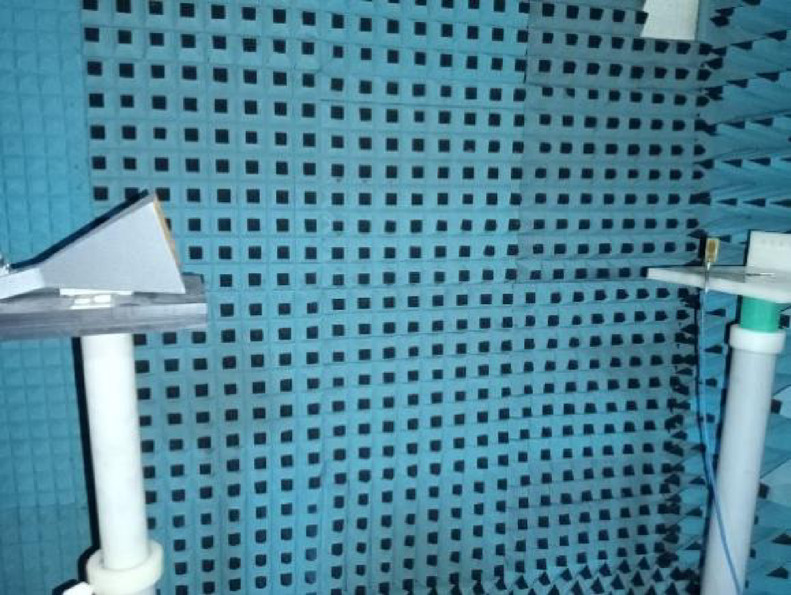
AUT in anechoic chamber with a test distance of 1.5 m.

Radiation patterns are measured in anechoic chamber. The Antenna Under Test (AUT) was connected to VNA and mounted on a precise rotation stage via a low – loss cable. The standard criteria to satisfy the far – field condition is R ≥ max ($\:2{D}^{2}/\lambda\:,\:10\lambda\:$) where D is the largest physical dimension of proposed antenna. On satisfying this condition a test distance of 1.5 m is there between the AUT and horn antenna which is shown in the Fig. 18.

**Fig. 19:**
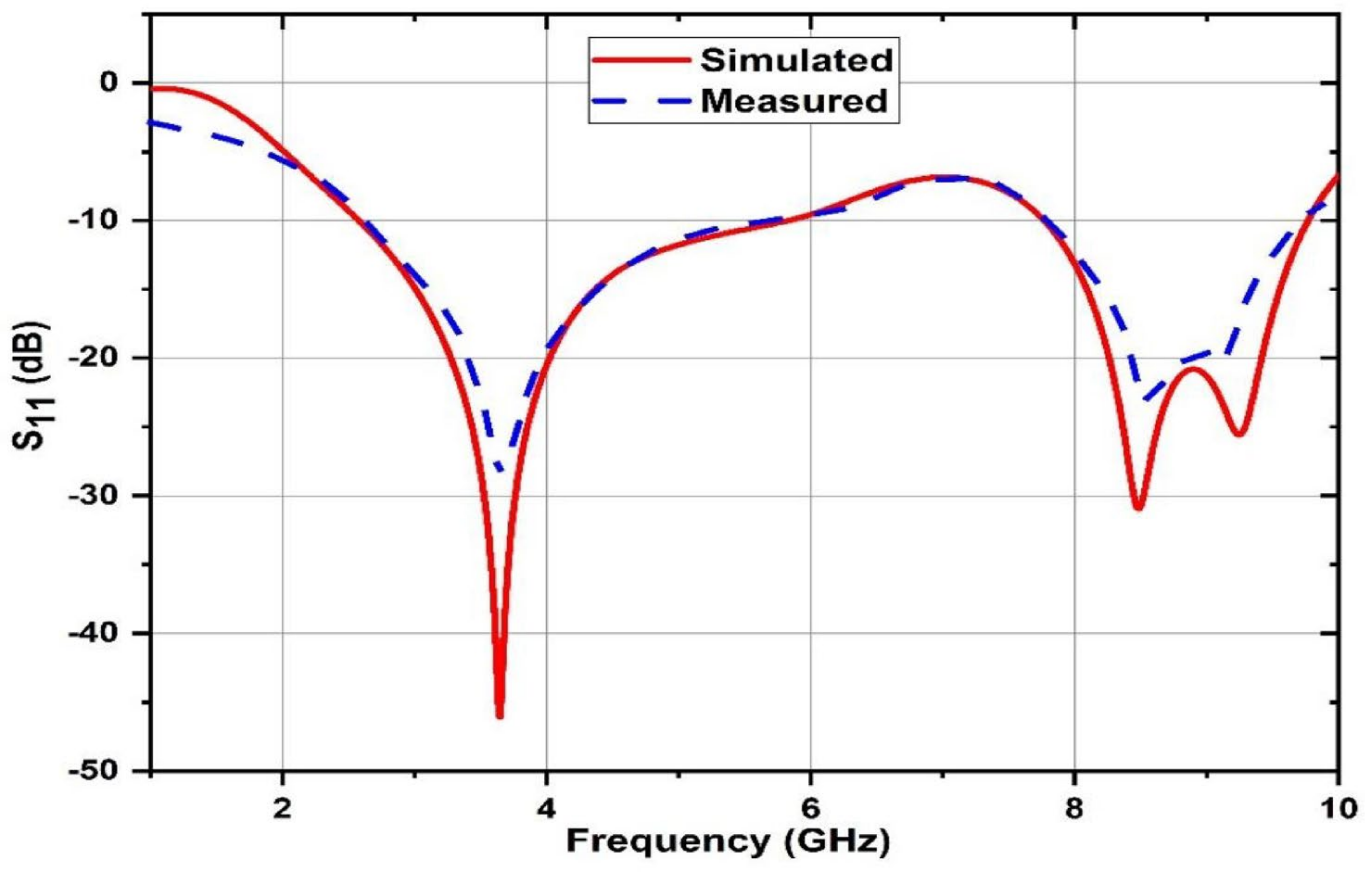
Simulated and measured results obtained for S_11_.

Figure 19. shows the fabricated prototype operating at three different frequencies with two dual broad band frequencies. The propounded antenna is operating at three distinct frequencies such as 3.64 GHz, 8.49 GHz and 9.25 GHz with a magnitude of −46.01dB, −30.9dB and − 25.59dB, respectively. Both the simulated and measured results are compared, and the fabricated prototype exhibits a decent performance matching the simulated antenna which is shown in the figure below.

**Fig. 20:**
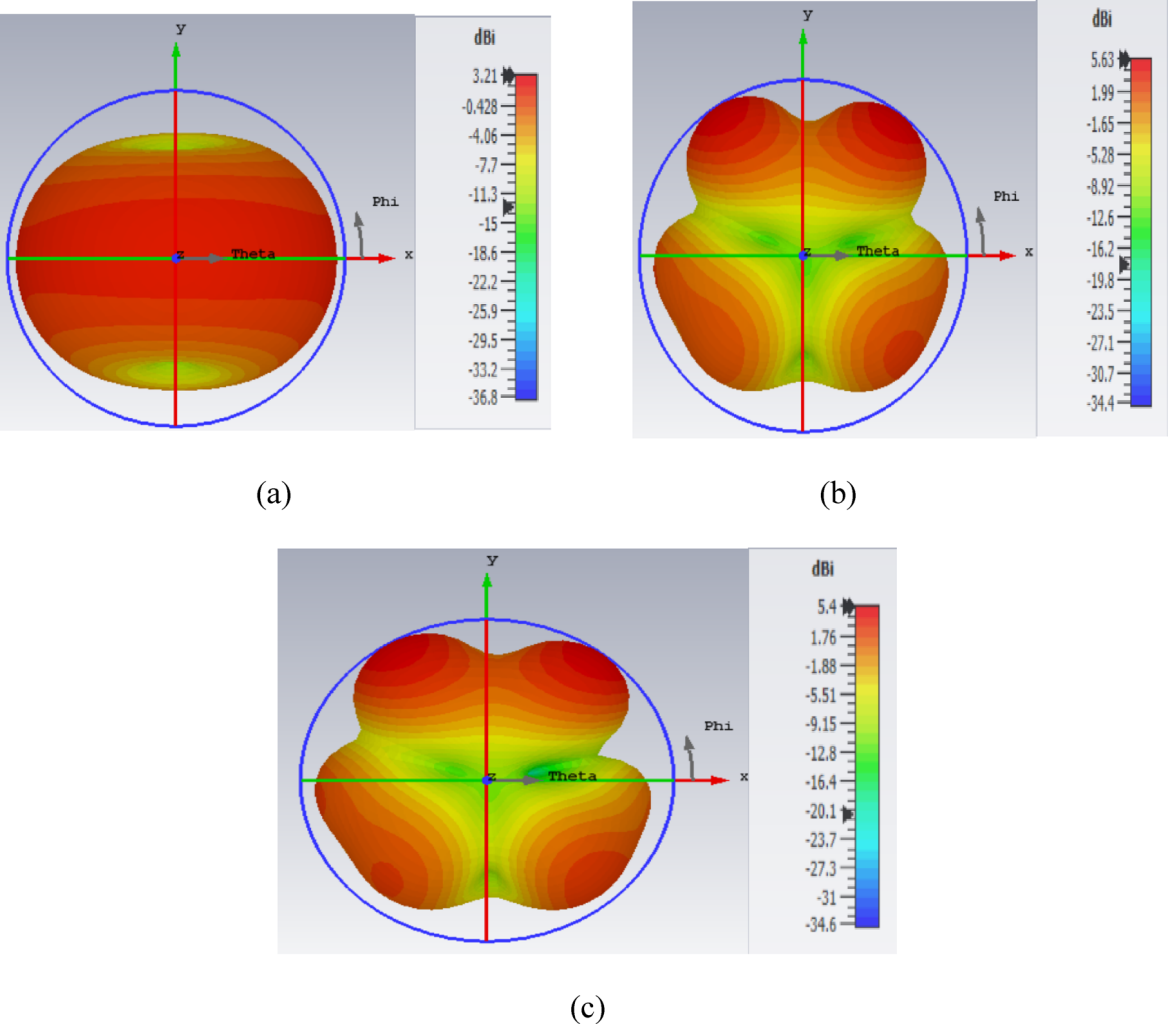
Obtained gains at the corresponding resonating frequencies of (**a**) 3.64 GHz (**b**) 8.49 GHz (**c**) 9.25 GHz.

**Fig. 21:**
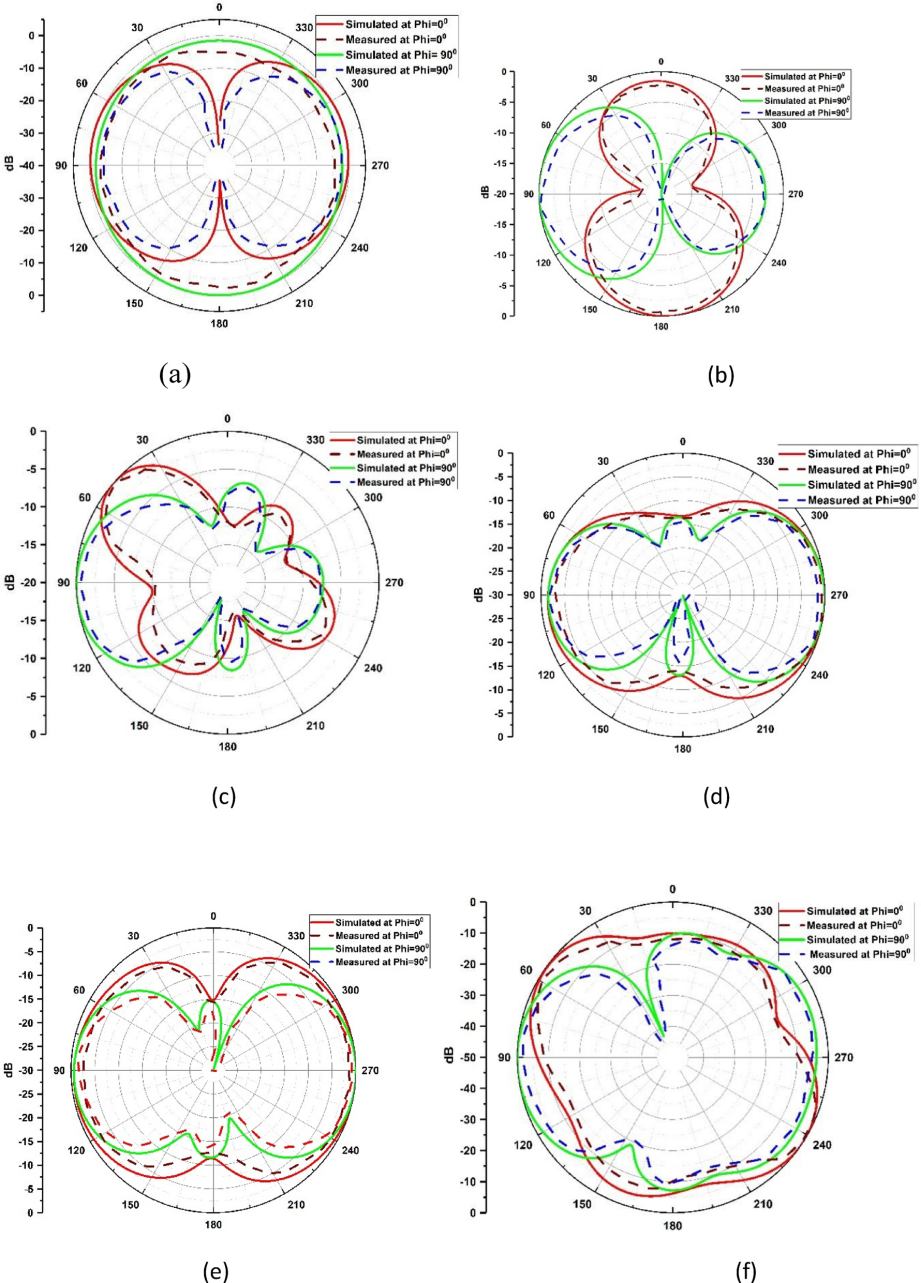
Co-pol and cross-pol radiation patterns (**a**) 3.64 GHz E-Plane (**b**) 3.64 GHz H-plane (**c**) 8.49 GHz E-Plane (**d**) 8.49 GHz H-Plane (**e**) 9.25 GHz E-Plane (**f**) 9.25 GHz H-Plane.

Figure 20. shows the CST generated three dimensional (3D) graphs for the three distinct operating frequencies. Later, the simulated co and cross polarized E and H plane are experimented by comparing it with the measured results and are represented in the Fig. 21 (a – f).

This Fig. 20 shows the three-dimensional (3D) gain at the corresponding resonating frequencies i.e., 3.64 GHz, 8.49 GHz and 9.25 GHz with a gain of 3.21dBi, 5.63dBi and 5.4dBi, respectively.

**Table 4:** Simulated and measured gain of the proposed antenna.

Resonant frequency (GHz)	Simulated gain (dBi)	Measured gain (dBi)	Δ (dB)
3.64	3.21	2.81	0.40
8.49	5.63	4.94	0.69
9.25	5.40	4.74	0.66

The Table 4 gives the comparison between the simulated and measured gain values at three different resonating frequencies of the proposed antenna. It shows that the measured values are slightly lower than the simulated values and those deviations are due to fabrication tolerances, chamber calibration uncertainties and substrate losses.

**Fig. 22:**
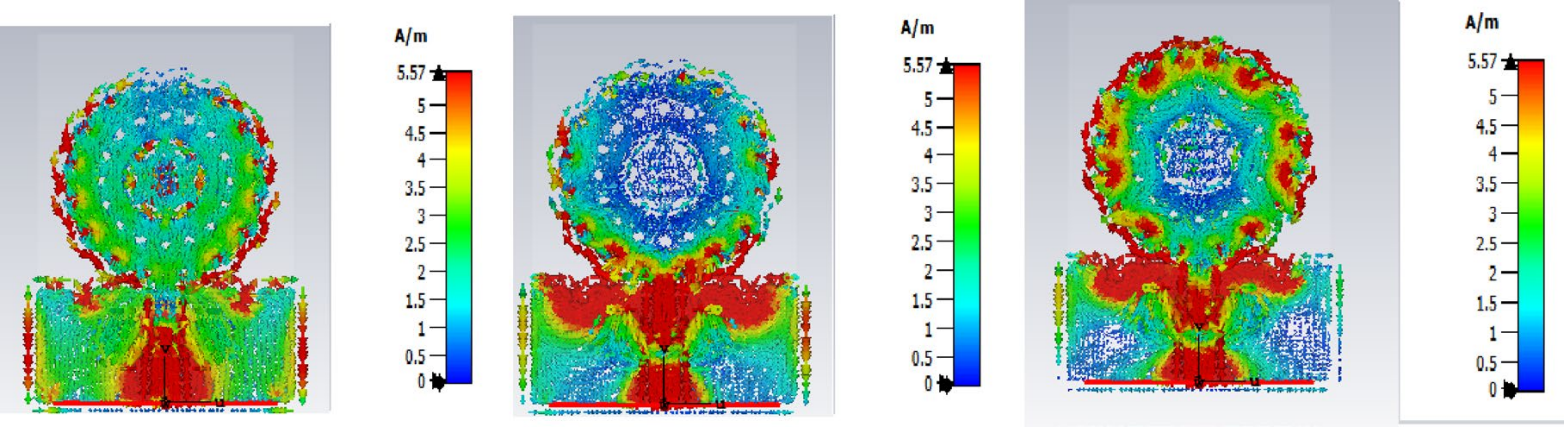
Surface current distributions at resonating frequencies (**a**) 3.64 GHz (**b**) 8.49 GHz (**c**) 9.25 GHz.

Figure 22 shows the surface current distributions at the three different resonating frequencies. At the first resonating frequency 3.64 GHz, the current distribution is only confined to the feed and a little bit in the radiating patch and is flowing uniformly which shows that it is the fundamental one and the corresponding radiation pattern is omnidirectional characteristic. For the second resonating frequency 8.49 GHz, the current distributed and non uniform and extending over partial plane ground which indicates the high order harmonic mode. At the third resonating frequency 9.25 GHz the current distribution is further extended to the radiating patch and fractal edges. The current distribution of the proposed prototype at the resonating frequencies states that the antenna exhibits the fundamental mode at the first resonating frequency and harmonic mode at the other two frequencies.

**Fig. 23:**
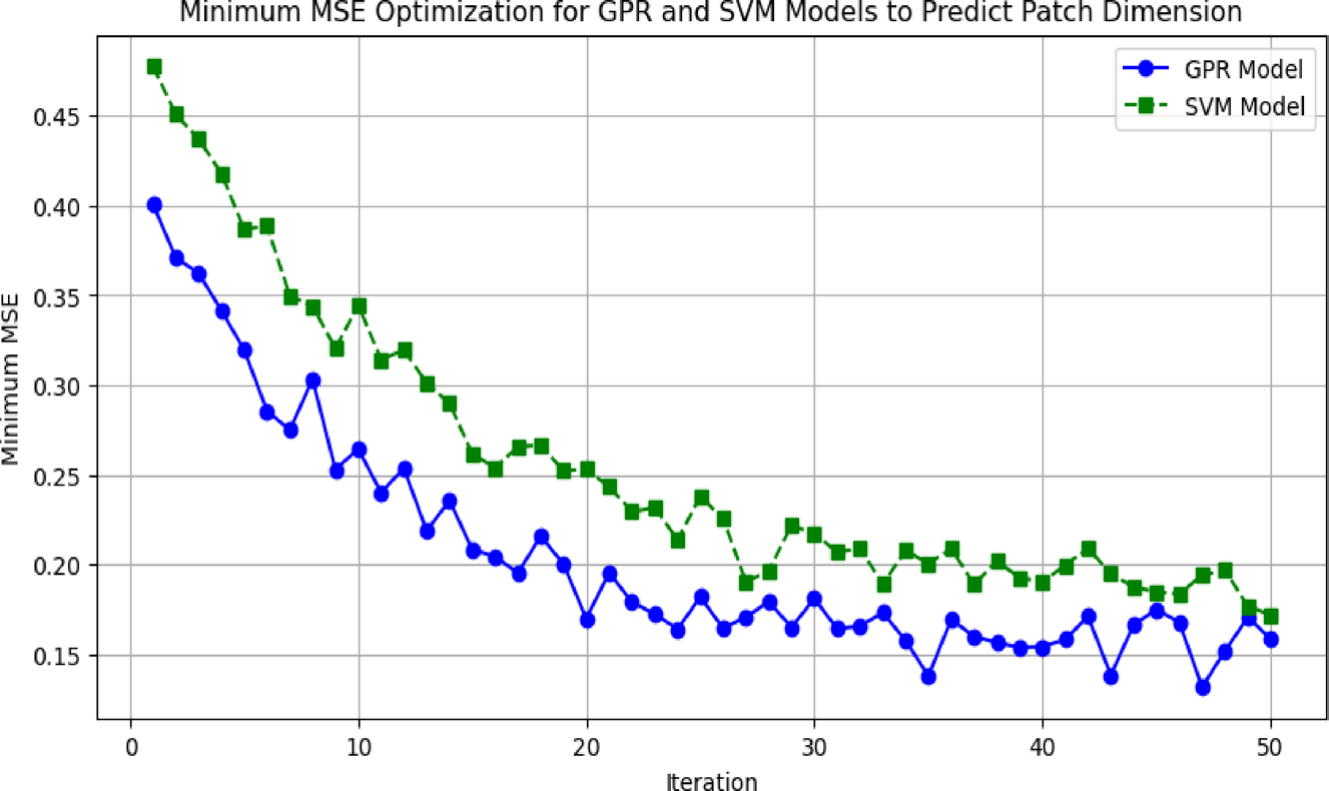
Minimum MSE optimization for GPR and SVM models to predict patch dimension.

In Fig. 23 we compare the iterative performance of GPR and SVR models in minimizing Mean Squared Error (MSE) for patch dimension prediction n. GPR model (blue line) has a decreasing MSE that is smooth and consistent which ultimately converges to an optimal solution, while SVR model (green dashed line) is more oscillatory and requires further fine tuning to stabilize. It is evident that the GPR model consistently outperforms the SVR model in terms of lower MSE values that indicates its better ability to deal with complicated, nonlinear relationships between design parameters and target variables.

**Fig. 24:**
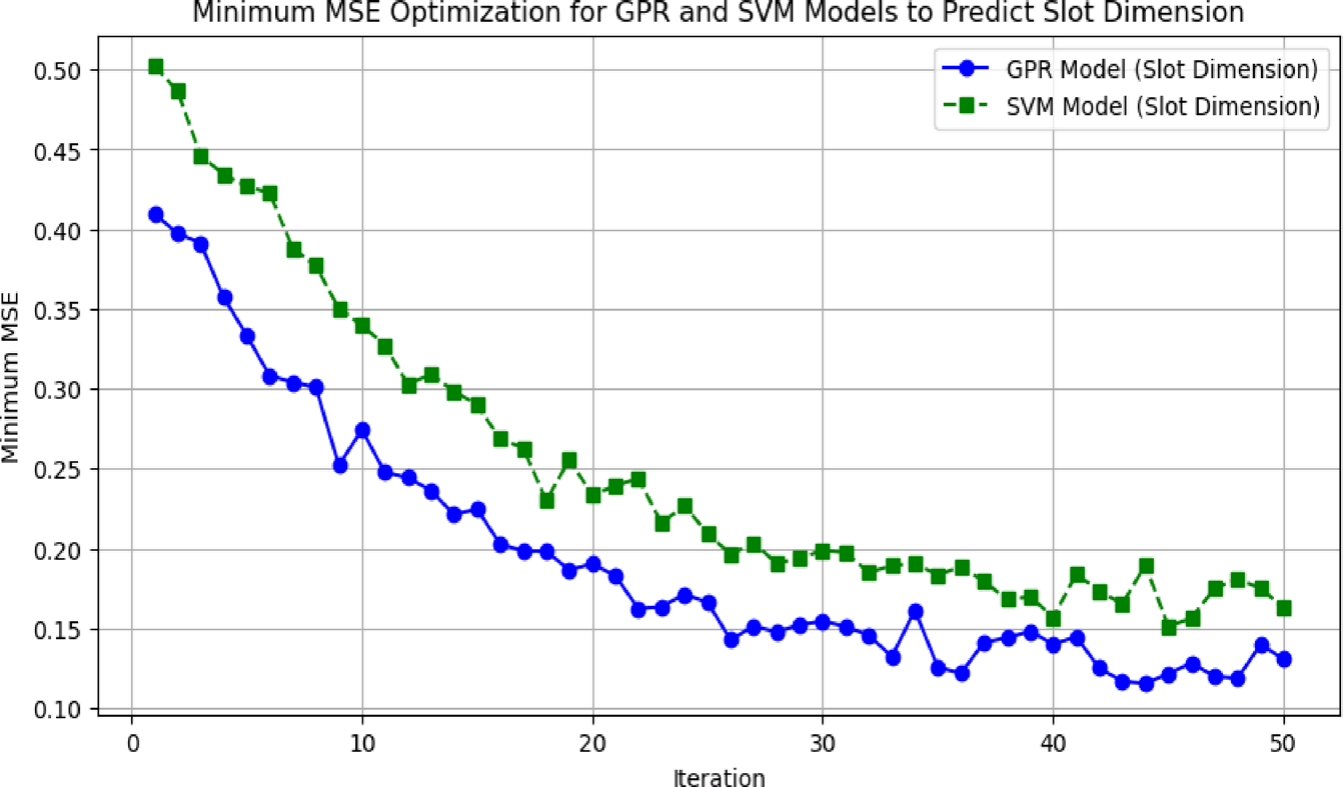
Minimum MSE optimization for GPR and SVM models to predict slot dimension.

The convergence behavior of GPR and Support Vector Machine (SVM) models in minimizing Mean Squared Error (MSE) for slot dimension prediction over 50 iterations is shown in Fig. 24.

Finally, the GPR model shown (blue line) has a stable and smooth decrease of MSE towards a smaller error value, which shows that it is capable of high prediction and has excellent efficiency to deal with complex and nonlinear relationship. On the other hand, the SVM model, shown by the green dashed line in the optimization trajectory, presents a bumpier optimization trajectory with few increases of error, indicating less stable process of convergence. Both models reduce MSE over iterations and GPR outperforms SVM with a much lower error margin in predicting slot dimensions for antenna design application.

**Fig. 25:**
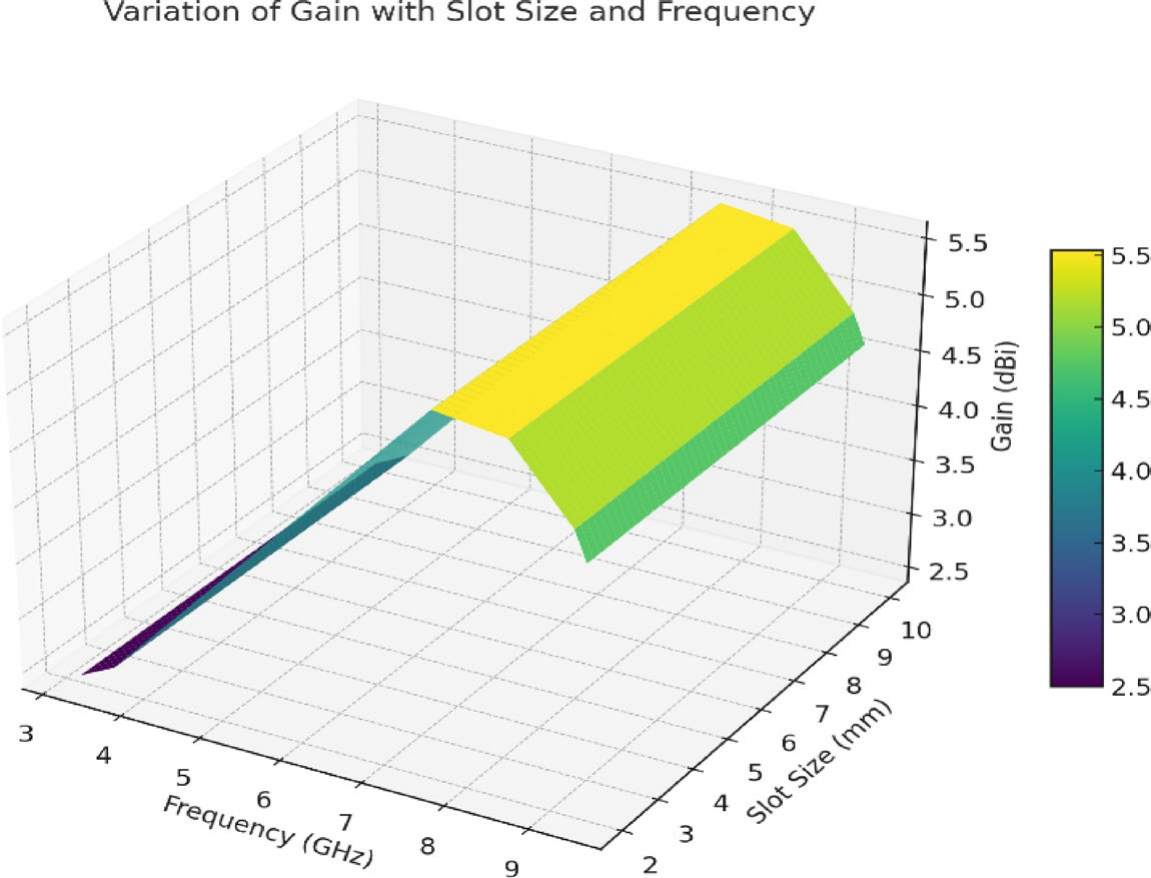
Variation of gain with slot size and resonant frequency.

The relationship between the slot size, the resonant frequency, and the gain in the designed antenna system is shown in Fig. 25. The graph definitively shows how the values of gain change due to changes in slot size (measured in mm) and resonant frequency (measured in GHz). The surface peaks represent the combinations of slot size and resonant frequency where the antenna achieves maximum gain, whereas the valleys represent lower gain configurations. The purpose of this visualization is to show how slot size and resonant frequency must work together to tune the antenna’s performance and determine the best design parameters for higher efficiency and radiation characteristics. With this analysis, engineers can see which slot sizes will offer the best gain during the process of assembling the antenna.

**Fig. 26:**
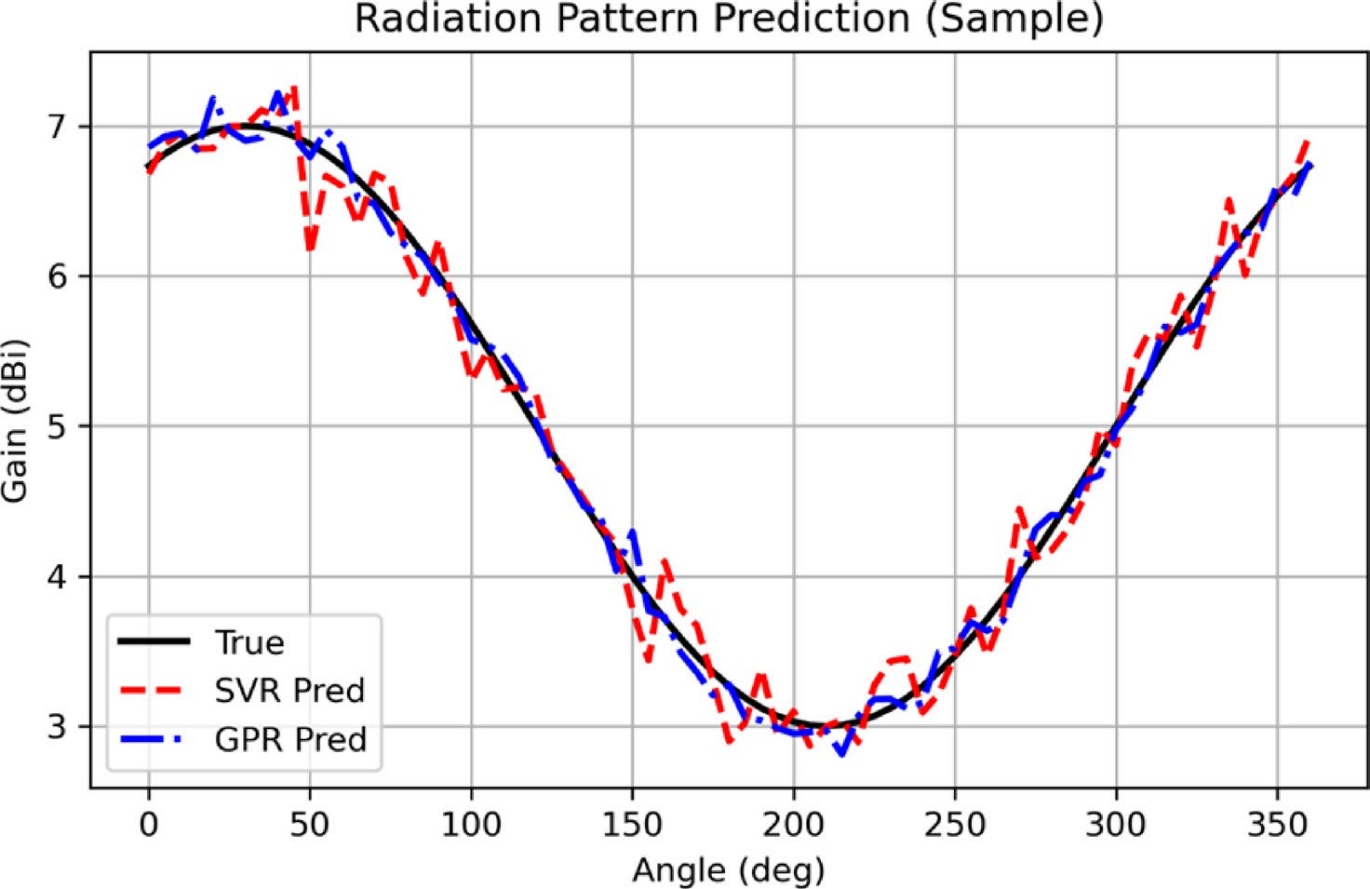
Radiation pattern prediction using ML Algorithms.

**Fig. 27:**
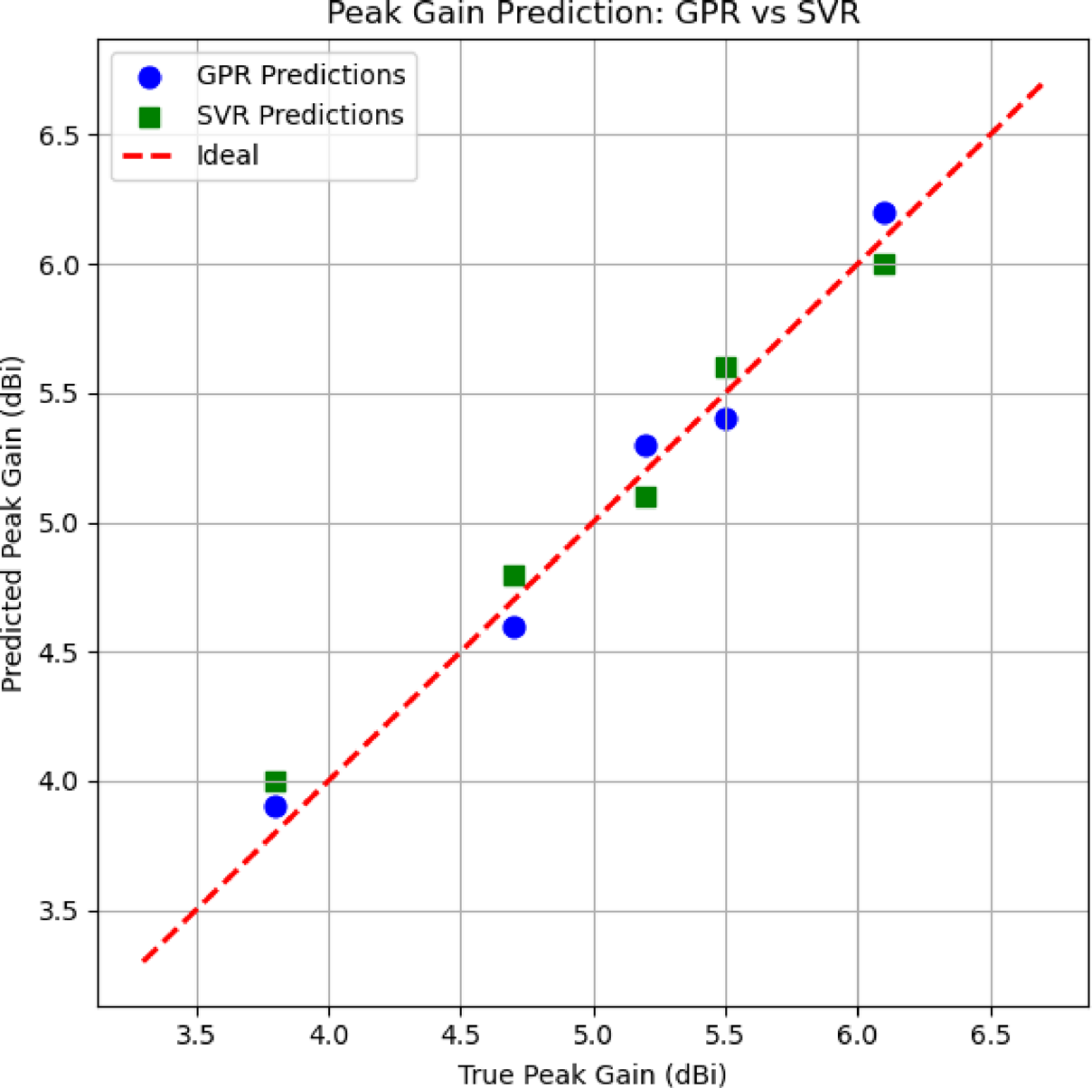
Gain prediction using ML Algorithms.

Figure 26 shows the radiation patterns that are predicted using ML algorithms. Blue dashed line shows for GPR whereas the red dashed line is for SVR. The plain black is the simulated radiation pattern of the proposed antenna. Figure 27 shows the gain that was predicted using ML algorithm. The red dashed line is the simulated gain, green boxes resembles SVM and blue dots are for GPR.

**Table 5:** Tabulated results of GPR and SVM models.

Parameter	GPR Model	SVR Model
Mean Squared Error (MSE)	0.15	0.20
R-Squared (R2R^2R2)	0.98	0.95
Convergence Time (s)	12.5	10.3
Predicted Resonant Frequency (GHz)	2.45 ± 0.05	2.46 ± 0.1
Predicted Slot Dimension (mm)	9.8 ± 0.39	10.2 ± 0.5
Predicted Patch Dimension (mm)	28.5 ± 0.5	28.7 ± 0.7
Prediction Stability (%)	98.5	95.8
Training Dataset Size	3822 Samples	3822 samples

GPR and SVR models are compared based on their suitability in antenna design optimization and their strengths are highlighted. It was found that the GPR model was a highly reliable model to predict resonant frequency (2.45 ± 0.05 GHz), slot dimension (9.8 ± 0.3 mm) and patch dimension (28.5 ± 0.5 mm) with lower Mean Squared Error (MSE) of 0.15 and higher 2 score of 0.98. In addition, GPR also provided uncertainty quantification, allowing us to provide useful confidence intervals in design optimization as shown in Table 5. They achieved lower accuracy (MSE = 0.20, 2 = 0.95) but converged faster (10.3 s vs. 12.5 s for GPR and are thus well suited to rapid, deterministic predictions. Simulated and measured 11 responses and radiation patterns of both models agreed sufficiently to validate their use in antenna design workflows. For complex design with high accuracy, GPR is used; for less complex design with faster computation but moderate accuracy, SVR is applied.

**Table 6:** Comparison of antenna performance before and after ML optimization.

Parameter	Before ML Optimization	After ML Optimization
Resonant Frequencies (GHz)	1.15, 3.75	1.22, 3.82
Return Loss (dB)	−12.4, −13.1	−22.8, −18.5
Bandwidth (MHz)	180, 240	320, 350
Peak Gain (dBi)	1.5, 2.1	3.3, 4.0
Efficiency (%)	62	87

Table 6 shows the clear difference in antenna performance before and after processing of data using ML methods based on GPR and SVR. At the beginning, the antenna showed resonances at 1.15 GHz and 3.75 GHz, but these shifted slightly to 1.22 GHz and 3.82 GHz after optimization, proving that the antenna is now tuned more precisely. It was noticed that return loss improved greatly: from − 12.4 dB and − 13.1 dB to − 22.8 dB and − 18.5 dB, confirming more effective resistor matching. The larger bandwidth made it possible for the antenna to cover a bigger range of frequencies than before. Also, the peak gain increased from 1.5 to 2.1 dBi to 3.3 and 4.0 dBi, indicating that the radio now transmits with more power. By boosting efficiency from 62% to 87%, there were fewer power losses and better radiation performance.

### ML based performance prediction

Table 7 below gives a summary of the performance of the six different ML algorithms that are used for antenna parameter prediction. Out of which GPR has the highest accuracy with least MSE and MAE but it has high testing time due to its kernel function. KNN and DT have least accuracy than others which highlights its limitations for complex antenna modelling. ANN has less accuracy than compared with GPR, but it has fast testing time which is reliable for real time applications. Overall GPR will be efficient algorithm for predicting S – Parameters, gain and radiation patterns in this work.

**Table 7:** Comparison of performance of different ML algorithms.

Algorithm	Training time (s)	Testing time (s)	Accuracy (%)	MSE	MAE
SVM	2.4	0.11	95.5	0.21	0.11
GPR	4.9	0.21	97.9	0.13	0.08
ANN	6.1	0.04	96.3	0.15	0.09
KNN	1.2	0.32	93.4	0.21	0.14
RF	3.5	0.11	97.1	0.17	0.09
DT	0.95	0.05	92.5	0.29	0.16

The Table 8 represents the error in gain prediction and radiation pattern correlation along with the S_11_ error of different ML algorithms. Among them GPR provides the lowest gain and S_11_ error and highest radiation pattern correlation, and it is followed by ANN and RF. KNN and DT have highest error than SVM. Finally, for accurate prediction of gain and radiation patterns GPR will be the most reliable algorithm.

**Table 8:** Gain error and radiation pattern correlation of ML algorithms.

Algorithm	Gain error %	Radiation pattern Corr. (%)	S_11_ error %
SVM	3.1%	95.1%	4.1%
GPR	1.7%	98.7%	2.0%
ANN	2.4%	95.8%	2.7%
KNN	3.6%	93.5%	4.4%
RF	2.6%	95.0%	3.3%
DT	5.3%	92.2%	6.0%

## Conclusion

GPR and SVR models are compared and analyzed in detail to optimize compact microstrip antenna design in this paper. Results confirm the feasibility of machine learning driven methodologies to antenna design, leading to a dramatic reduction in computational effort while retaining high prediction accuracy and performance reliability. The GPR model demonstrated exceptional prediction accuracy, achieving a Mean Squared Error (MSE) of 0.15 and a score of 0.98. This was a good choice for optimizing nonlinear and complex antenna design parameters like resonant frequency (GHz), slot dimensions (mm), and patch dimensions (mm) due to its ability to quantify uncertainty, which provided useful insight into the reliability of predictions. Including confidence intervals allowed the parameter refinement to follow a data driven approach and achieve multiband operation with minimal return loss with precision.

However, the SVR model, with MSE of 0.20 and, provided deterministic predictions that converged faster (10.3 s versus 12.5 s for GPR. However, SVR did not include uncertainty quantification and was able to work with large datasets, offering reliable predictions that were appropriate for high throughput scenarios that demand rapid assessments. The multiband performance of the optimized antenna design showed excellent agreement between simulation and measurement, especially in response and radiation pattern, thus validating the accuracy of the proposed methodologies of machine learning-driven methodologies in antenna design, significantly reducing computational effort while ensuring high prediction accuracy and performance reliability. The comparison of GPR and SVR shows that they are complementary. In high precision and reliable scenarios, GPR performs better than SVR, but in other scenarios SVR gives faster predictions with acceptable accuracy. These results highlight the potential for the integration of machine learning with antenna design to accelerate design space exploration, optimization of key parameters, and the improvement of performance metrics. Future work will include augmenting the proposed framework with more powerful deep learning models like CNNs and Transformers for more complex geometries, across a wider frequency band. Furthermore, we will also focus on real-time implementations of machine learning assisted design methodologies to bridge the gap between simulations and practical applications in the next generation wireless communication systems. Research in the future may include using CNN or transformer models to quickly adjust the parameters of antennas in real time.

## Data Availability

The data used to support the findings of this study are included in the article.
